# Additional mitochondrial DNA influences the interactions between the nuclear and mitochondrial genomes in a bovine embryo model of nuclear transfer

**DOI:** 10.1038/s41598-018-25516-3

**Published:** 2018-05-08

**Authors:** Kanokwan Srirattana, Justin C. St. John

**Affiliations:** 1 Centre for Genetic Diseases, Hudson Institute of Medical Research, Clayton, VIC 3168 Australia; 2 Department of Molecular and Translational Sciences, Monash University, Clayton, VIC 3168 Australia

## Abstract

We generated cattle embryos using mitochondrial supplementation and somatic cell nuclear transfer (SCNT), named miNT, to determine how additional mitochondrial DNA (mtDNA) modulates the nuclear genome. To eliminate any confounding effects from somatic cell mtDNA in intraspecies SCNT, donor cell mtDNA was depleted prior to embryo production. Additional oocyte mtDNA did not affect embryo development rates but increased mtDNA copy number in blastocyst stage embryos. Moreover, miNT-derived blastocysts had different gene expression profiles when compared with SCNT-derived blastocysts. Additional mtDNA increased expression levels of genes involved in oxidative phosphorylation, cell cycle and DNA repair. Supplementing the embryo culture media with a histone deacetylase inhibitor, Trichostatin A (TSA), had no beneficial effects on the development of miNT-derived embryos, unlike SCNT-derived embryos. When compared with SCNT-derived blastocysts cultured in the presence of TSA, additional mtDNA alone had beneficial effects as the activity of glycolysis may increase and embryonic cell death may decrease. However, these beneficial effects were not found with additional mtDNA and TSA together, suggesting that additional mtDNA alone enhances reprogramming. In conclusion, additional mtDNA increased mtDNA copy number and expression levels of genes involved in energy production and embryo development in blastocyst stage embryos emphasising the importance of nuclear-mitochondrial interactions.

## Introduction

Mitochondria are membrane-bound organelles that generate the vast majority of cellular adenosine triphosphate (ATP) through oxidative phosphorylation (OXPHOS), which is conducted in the electron transfer chain. Mitochondria house the mitochondrial genome (mtDNA), which is approximately 16.3 kb in size in cattle^1^. The maternally inherited mammalian mitochondrial genome^2^ encodes 13 protein-encoding genes of the electron transfer chain and 22 tRNAs, two rRNAs and has one non-coding region, the D-loop, which contains two hypervariable regions^1^. As the other proteins of the electron transfer chain and the mitochondrion are encoded by the nuclear genome, the coordinated expression of genes from both the mitochondrial and nuclear genomes is essential for maintaining normal mitochondrial and cellular function^3,4^. Furthermore, individual cell types have distinct numbers of mitochondria and copies of mtDNA that are indicative of a cell’s requirement for ATP generated through OXPHOS (reviewed in^5–7^).

Oocytes with low mtDNA copy number and insufficient levels of ATP are more likely to fail to fertilise or arrest during preimplantation development and, thus, fail to develop to the blastocyst stage^8–13^. Ooplasmic and mitochondrial transfer, which involve the transfer of either oocyte cytoplasm or mitochondria into the oocyte, have been used to increase the probability of successful developmental outcome in many species such as mouse^14–21^, pig^11,12^, cattle^22–24^, and human^25–27^. Indeed, the supplementation of developmentally incompetent oocytes possessing low mtDNA copy number with approximately 800 copies of genetically identical mtDNA, as intracytoplasmic sperm injection is performed, can increase fertilisation outcome^12^ and development to the blastocyst stage^11^, as demonstrated in a porcine model. In this instance, developmental competence was assessed using the stain, brilliant cresyl blue (BCB) whereby oocytes that no longer express glucose-6-phosphate dehydrogenase (G6PDH) are developmentally competent and cannot breakdown the stain and, thus, label positively (BCB^+^). However, developmentally incompetent oocytes stain negatively (BCB^−^), as they continue to express G6DPH and breakdown the label^28^. BCB^−^ oocytes have significantly fewer copies of mtDNA (<50, 000) than BCB^+^ oocytes (>150, 000) and have lower fertilisation and developmental rates^11,12^. Mitochondrial supplementation of BCB^−^ oocytes also resulted in enhanced chromosomal gene expression patterns at the blastocyst stage ensuring that key developmental gene networks and pathways were more appropriately established^11^. Whilst there are benefits from supplementation of developmentally incompetent oocytes, it is unclear how supplementation would affect oocytes that have already achieved developmental competence and possess the appropriate numbers of mtDNA copy to support subsequent developmental events.

Somatic cell nuclear transfer (SCNT) is an assisted reproductive technology that involves the transfer a donor cell into a recipient oocyte, which has had its nuclear genome removed^29^. It provides an important tool for studying the interactions between the nuclear and mitochondrial genomes in embryos that are genetically identical. However, in SCNT, the mitochondria of the donor cell are also transferred to the oocyte. Somatic cell mitochondria can initiate key cellular signalling processes including apoptosis (reviewed in^30^) and have negative effects on embryo development^15,19^. In addition, somatic cell mtDNA is likely to have accumulated deletions or mutations as part of the aging process^31,32^, which, if preferentially replicated during development, could adversely affect the health of the offspring.

Donor cell mtDNA can be eliminated prior to intraspecies SCNT by using mtDNA depletion agents, such as ethidium bromide or 2′,3′-dideoxycytidine (ddC). This approach ensures that the resultant embryo transmits recipient oocyte-only mtDNA^33–35^ and, thus, mimics the patterns of mtDNA inheritance consistent with the less invasive assisted reproductive technologies, such as *in vitro* fertilisation^36^, and natural conception^2^. ddC is an effective depletion agent as it directly interferes with mtDNA replication^37^ and does not affect the integrity of the cell’s chromosomal DNA as the depletion process takes place^35^. However, for interspecies SCNT, which uses a donor cell and an oocyte from two different species, and is widely used to rescue endangered species^38–44^, there may be benefits in retaining donor cell mtDNA. This would enable more closely related nuclear and mitochondrial genomes to interact more efficiently^45,46^. Indeed, it has been previously argued that there is a relationship between the mtDNA genetic distance between the donor cell and the recipient oocyte for successful SCNT especially as increased mtDNA genetic distance results in early developmental failure^33,47,48^.

In this work, we have generated embryos using developmentally competent cattle oocytes and mtDNA depleted somatic cells with mitochondrial supplementation, a process referred to as miNT, to determine the effects of increasing mtDNA copy number on the interactions between the nuclear and mitochondrial genomes during early development. As Trichostatin A (TSA), a histone deacetylate inhibitor, has been used to improve SCNT efficiency in many species (reviewed in^49^), we have also used TSA to determine whether developmental outcome could be further enhanced.

We show that the addition of mtDNA to developmentally competent oocytes did not affect the developmental potential of miNT-derived embryos. However, miNT-derived blastocyst stage embryos had significantly increased mtDNA copy number compared with SCNT-derived blastocysts. TSA did not enhance blastocyst rates or modulate gene expression patterns of miNT-derived embryos unlike non-supplemented oocytes. Notably, miNT-derived embryos had different gene expression patterns compared with SCNT-derived embryos. To this extent, the additional mtDNA upregulated expression levels of genes associated with cellular energy production. Moreover, depleting donor cell mtDNA increased the levels of expression of genes involved in embryonic development in miNT-derived embryos.

## Results

### Developmental potential of embryos produced by miNT and SCNT

To determine the developmental potential of introducing additional mtDNA into developmentally competent oocytes, we generated a series of embryos using SCNT. We selected for developmental competence using BCB. A proportion of BCB^+^ oocytes were also supplemented with additional copies of oocyte mtDNA (563.1 ± 57.7, mean ± SEM) as SCNT was performed (miNT). The SCNT embryos were previously reported^50^ but were generated at the same time point as the miNT embryos. For each round of embryo production, parthenogenetically activated (PA) embryos were also generated and used as the control group. In all, four replicates were generated for each treatment. When nondepleted cells were used as the donor cells, the rates of fusion, cleavage and development to the blastocyst stage of miNT-derived embryos (93.3%, 90.7% and 38.5%, respectively) were not significantly different when compared with SCNT-derived embryos without mitochondrial supplementation (93.7%, 87.1% and 35.9%, respectively, Table 1). When using depleted cells as the donor cells, the fusion rates following miNT were lower than for nondepleted cells generated through either SCNT (76.5% and 93.7%, respectively) or miNT (82.7% and 93.3%, respectively, P < 0.05). Moreover, there were no significant differences in the rates of cleavage and development to the blastocyst stage between embryos produced by miNT and SCNT using depleted cells (blastocyst rates: 20.2% and 20.1%, respectively). In addition, there was only a marginal benefit from using TSA on the developmental potential of miNT-derived embryos.

**Table 1:** Developmental potential of miNT and SCNT embryos generated from mtDNA depleted and nondepleted donor cells in the presence and absence of TSA.

Donor cell	Method	TSA	Fused (%)	No. embryo	Cleaved (%)*	No. (%)* embryo developed to		
						8-Cell	Morula	Blastocyst
Nondepleted	miNT	−	168/180 (93.3 ± 1.3)^a^	161	146 (90.7 ± 1.9)^ab^	103 (64.0 ± 8.9)	63 (39.1 ± 5.3)^ab^	62 (38.5 ± 5.7)^a^
	SCNT^†^	−	254/271 (93.7 ± 1.6)^a^	248	216 (87.1 ± 0.3)^ab^	152 (61.3 ± 2.2)	91 (36.7 ± 2.9)^ab^	89 (35.9 ± 3.4)^a^
Depleted	miNT	−	172/208 (82.7 ± 3.7)^b^	168	142 (84.5 ± 3.5)^ab^	75 (44.6 ± 1.7)	43 (25.6 ± 2.6)^bc^	34 (20.2 ± 2.0)^b^
	SCNT^†^	−	189/247 (76.5 ± 1.7)^b^	174	137 (78.7 ± 5.0)^b^	77 (44.3 ± 4.6)	36 (20.7 ± 3.4)^c^	35 (20.1 ± 3.8)^b^
	miNT	+	182/231 (78.8 ± 0.9)^b^	161	146 (90.7 ± 3.2)^a^	97 (60.2 ± 8.6)	44 (27.3 ± 2.0)^bc^	38 (23.6 ± 1.2)^b^
	SCNT^†^	+	173/230 (75.2 ± 3.1)^b^	163	144 (88.3 ± 3.6)^ab^	98 (60.1 ± 6.6)	58 (35.6 ± 5.0)^ab^	57 (35.0 ± 4.6)^a^
	PA	−	N/A	164	143 (87.2 ± 2.9)^ab^	93 (56.7 ± 9.2)	71 (43.3 ± 2.5)^a^	71 (43.3 ± 2.5)^a^

miNT, mitochondrial supplementation in combination with SCNT; SCNT: Somatic cell nuclear transfer;

PA: Parthenogenetic activation.

−, absence of TSA; +, presence of TSA; TSA, Trichostatin A.

*Percentages calculated from the number of embryos that cleaved and developed to each stage.

^†^Data from our previous report^50^. Values are mean ± SEM.

Four replicates per treatment.

Different superscripts within a column indicate significant differences (P < 0.05, ANOVA with Duncan’s multiple range test using SAS version 9).

### Analysis of mtDNA copy number and cell number in miNT- and SCNT-derived embryos at the blastocyst stage

**Figure 1:**
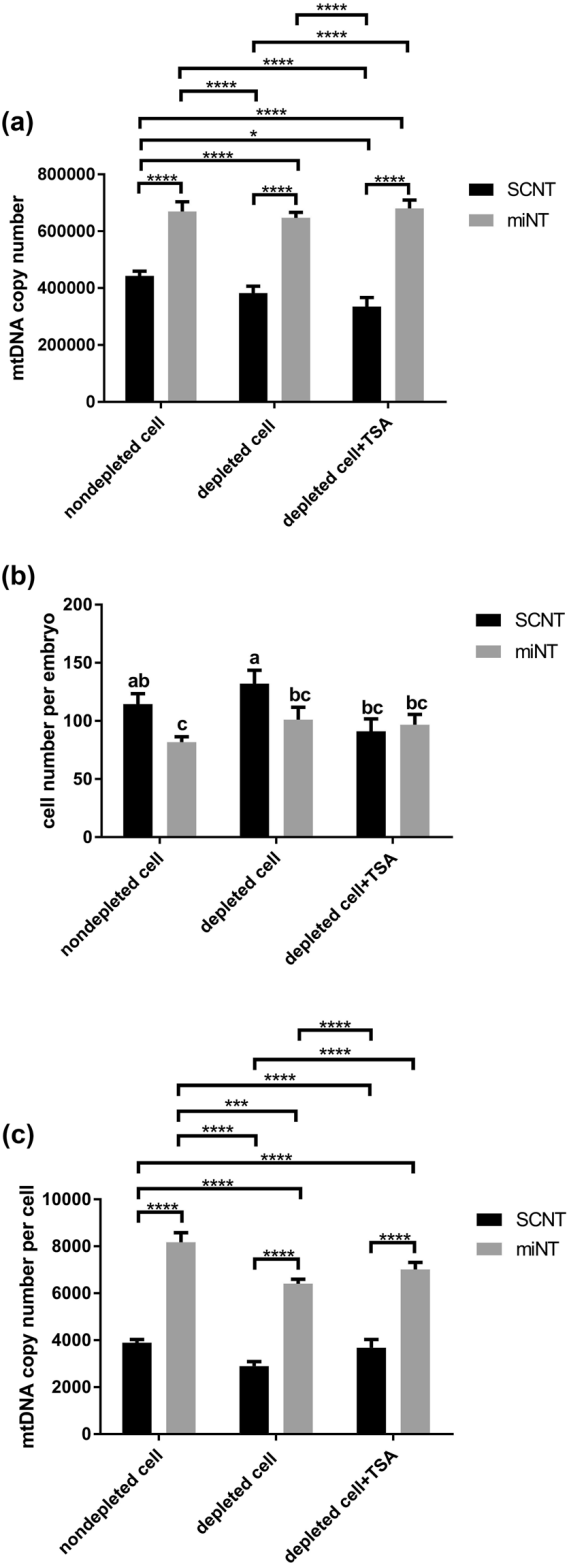
Assessment of cell number and mtDNA copy number in miNT- and SCNT-derived embryos at the blastocyst stage. (**a**) mtDNA copy number per embryo (n = 10/group). Mean values (±SEM) are shown. The differences were analysed for significance by two-way ANOVA with Sidak’s multiple comparisons test using GraphPad Prism version 6.01. *,****indicate P < 0.05 and P < 0.0001, respectively. (**b**) Cell number for miNT and SCNT embryos at the blastocyst stage (n = 3/group). Mean values (±SEM) are shown. Different superscripts within a column indicate significant differences (P < 0.05, ANOVA with Duncan’s multiple range test). (**c**) mtDNA copy number per cell (n = 10/group). Mean values (±SEM) are shown. The differences were analysed for significance by two-way ANOVA with Sidak’s multiple comparisons test. ***,****indicate P < 0.001 and P < 0.0001, respectively. SCNT-derived embryo data were obtained from our previous report^50^. The embryos were produced at the same time point.

To determine if mitochondrial supplementation affected the levels of mtDNA present in embryos at the blastocyst stage, assessment of mtDNA copy number and cell number were performed. MtDNA copy number for miNT-derived blastocysts was significantly higher (circa 1.5 to 2.0 times) than for SCNT blastocysts derived from nondepleted cells and depleted cells either in the absence or presence of TSA (P < 0.0001, Fig. 1a). There were no significant differences in mtDNA copy number for miNT-derived blastocysts generated from nondepleted cells (669, 758.4 ± 33, 667.4, mean ± SEM), depleted cells (646, 913.6 ± 19, 578.5) and depleted cells cultured in the presence of TSA (680, 408.9 ± 29, 064.9). On the other hand, mtDNA copy number for SCNT-derived blastocysts generated from nondepleted cells (442, 849.6 ± 16, 509.7) was significantly higher than for SCNT blastocysts derived from depleted cells cultured in the presence of TSA (334, 908.9 ± 31, 807.7, P < 0.05).

Cell numbers for SCNT-derived blastocysts were significantly higher than for miNT-derived blastocysts generated from nondepleted cells (114.3 ± 9.1 and 81.7 ± 4.8, mean ± SEM, respectively, P < 0.05, Fig. 1b) and depleted cells (132.0 ± 11.5 and 101.0 ± 10.8, respectively). However, no significant differences in cell number were found between SCNT (91.0 ± 10.8) and miNT (96.7 ± 9.0) blastocysts derived from depleted cells in the presence of TSA. Notably, depleting mtDNA from the donor cells or the use of TSA had no significant difference on cell number for miNT blastocysts.

In terms of mtDNA copy number per cell, levels were significantly higher in miNT-derived blastocysts than SCNT blastocysts derived from nondepleted cells and depleted cells either in the absence or presence of TSA (P < 0.0001, Fig. 1c). MtDNA copy number for miNT blastocysts derived from nondepleted cells (8,167.8 ± 410.6) was significantly higher than for depleted cells (6,405.1 ± 193.9, P < 0001), but not for depleted cells cultured in the presence of TSA (7,014.5 ± 299.6). On the other hand, there was no significant difference in mtDNA copy number per cell for SCNT blastocysts derived from nondepleted cells (3,884.7 ± 144.8), depleted cells (2,896.7 ± 187.2), and depleted cells cultured in the presence of TSA (3,680.3 ± 349.5).

### Analysis of differentially expressed genes in miNT- and SCNT-derived blastocyst stage embryos

To determine whether gene expression profiles of embryos were modulated by mitochondrial supplementation, RNA next generation sequencing of single miNT and SCNT-derived embryos was performed. The number of genes expressed in each blastocyst was 8, 072.8 ± 334.5 (mean ± SEM) for miNT blastocysts derived from nondepleted cells; 8, 040.5 ± 540.1 for miNT blastocysts derived from depleted cells; 8, 368.3 ± 381.7 for miNT blastocysts derived from depleted cells cultured in presence of TSA; 8, 767.7 ± 99.3 for SCNT blastocysts derived from depleted cells; and 8, 821.3 ± 107.3 for SCNT blastocysts derived from depleted cells cultured in the presence of TSA. There was no significant difference in the number of genes expressed amongst each group.

**Figure 2:**
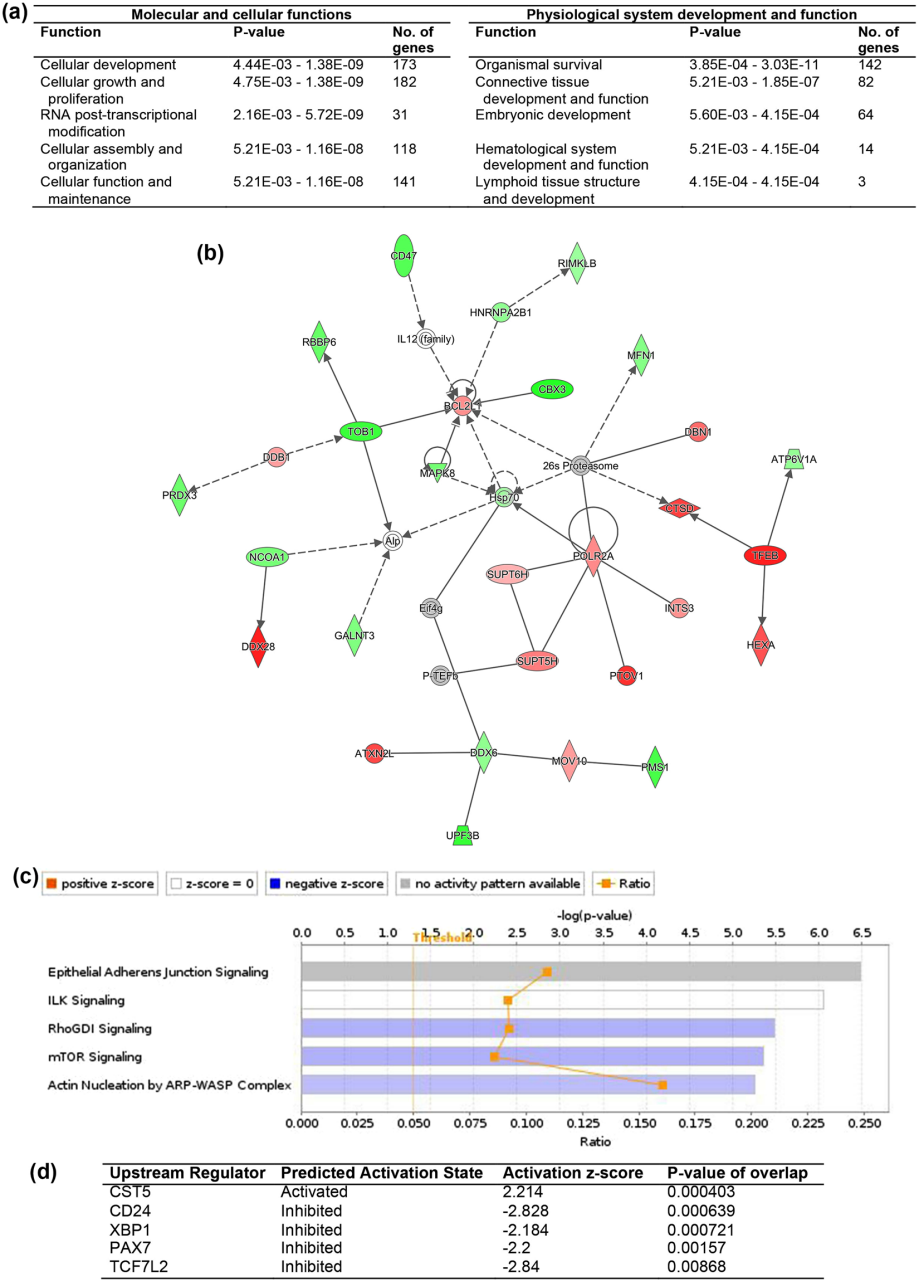
IPA analysis of DEGs from the comparison between miNT and SCNT blastocysts derived from depleted cells (n = 3 to 4/group). (**a**) List of the top five biological functions. (**b**) Gene interaction network involved in gene expression, cell morphology, and cellular function and maintenance. Upregulated and downregulated genes are represented in red and green, respectively. The intensity of colour represents the degree of regulation. Colourless or grey nodes are genes involved in the network which were obtained from the Ingenuity Knowledge Base. The solid and dashed lines represent direct and indirect interactions, respectively. (**c**) The top five statistically significant canonical pathways. (**d**) List of upstream regulators.

For the comparisons between miNT and SCNT blastocysts derived from depleted cells cultured in the absence of TSA, 188 differentially expressed genes (DEGs) were upregulated and 321 DEGs were downregulated in the miNT blastocysts (FDR < 0.05, Supplementary File 1). The top ten most significant DEGs are listed in Supplementary Table 1. Analysis of downstream effects was performed using Ingenuity Pathway Analysis (IPA) to identify biological activities influenced by the DEGs. The most affected molecular and cellular function was cellular development, whilst the top annotation for physiological system, development and function was organismal survival (Fig. 2a). The annotated functions with a significant activation z-score are listed in Supplementary Table 2. The formation of spindle apparatus and mitotic spindle, and recombination of cells were predicted to increase. Cell viability and movement, ubiquitination of protein, metabolism of DNA, and DNA replication and translation were predicted to decrease. The top five networks identified from the DEGs, which had IPA scores (-log(P-value)) ranging from 36 to 40, are listed in Supplementary Table 3. Fourteen upregulated DEGs and 15 downregulated DEGs were involved in gene expression, cell morphology, and cellular and maintenance functions (Fig. 2b). The most significantly affected canonical pathway was the ‘epithelial adherens junction signalling’ pathway. The top five canonical pathways are shown in Fig. 2c. Using upstream regulation analysis, CD24 and other three other transcription regulators (TCF7L2, PAX7 and XBP1) were predicted to be inhibited (|z-score| > 2, P < 0.01, Fig. 2d) whilst CST5 was predicted to be activated.

**Figure 3:**
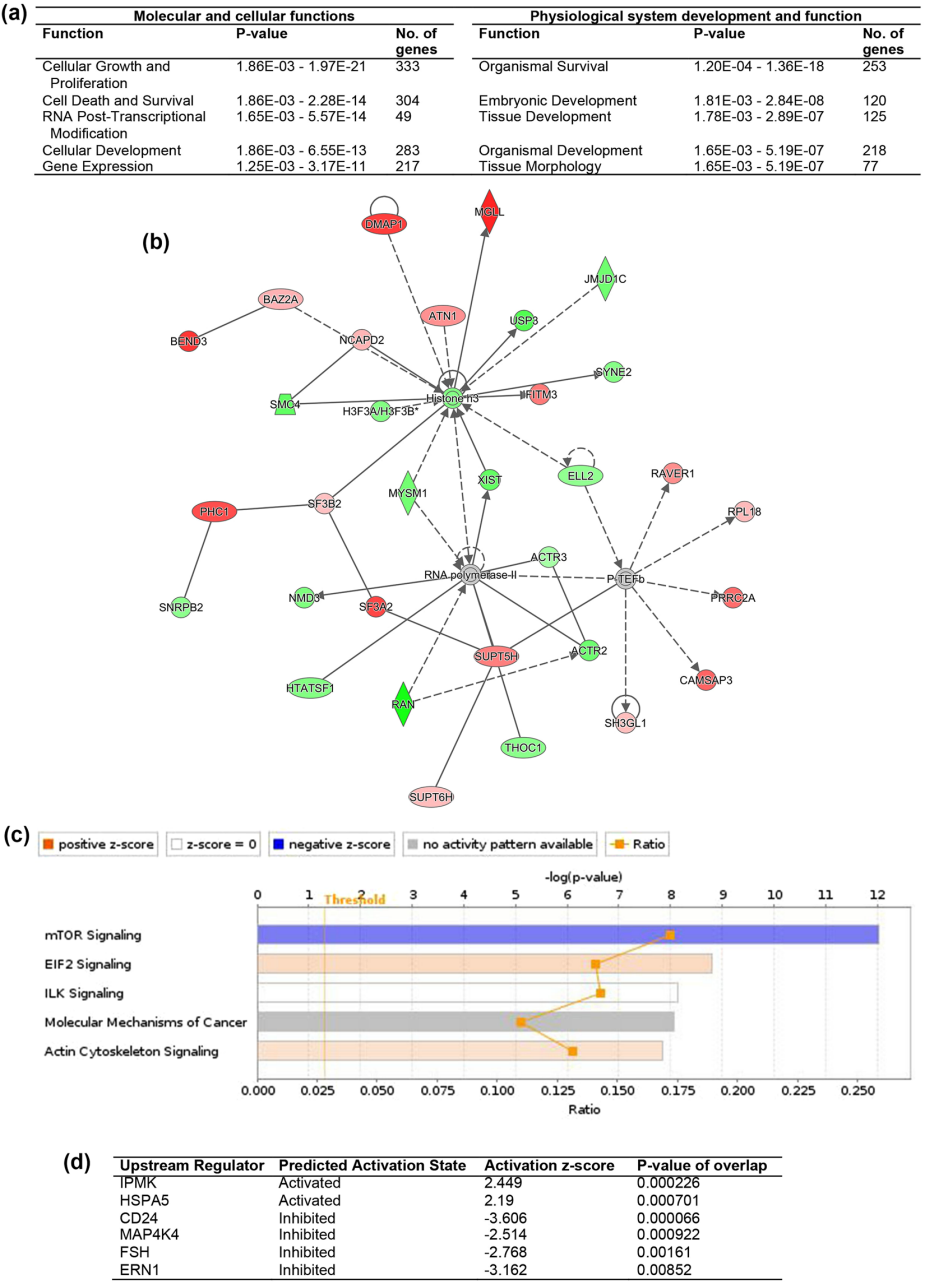
IPA analysis of DEGs from the comparison between miNT blastocysts derived from depleted cells cultured in the absence of TSA and SCNT blastocysts derived from depleted cells cultured in the presence of TSA (n = 3 to 4/group). (**a**) List of the top five biological functions. (**b**) Gene interaction network involved in DNA replication, recombination, and repair, gene expression, and cell cycle. Upregulated and downregulated genes are represented in red and green, respectively. The intensity of colour represents the degree of regulation. Colourless or grey nodes are genes involved in the network which were obtained from the Ingenuity Knowledge Base. The solid and dashed lines represent direct and indirect interactions, respectively. (**c**) The top five statistically significant canonical pathways. (**d**) List of upstream regulators.

Moreover, we compared expression patterns of miNT blastocysts derived from depleted cells cultured in the absence and presence of TSA to SCNT blastocysts derived from depleted cells cultured in the presence of TSA, which we assumed to be the gold standard, as we have previously demonstrated for SCNT embryos^35^. For the comparison between miNT blastocysts derived from depleted cells cultured in the absence of TSA and SCNT blastocysts derived from depleted cells cultured in the presence of TSA, 454 out of the 894 DEGs were upregulated (FDR < 0.05, Supplementary File 2). The top ten most significant DEGs are listed in Supplementary Table 1. From the IPA downstream effects analysis, the top significantly affected functions were cellular growth and proliferation, and organismal survival (Fig. 3a). Segregation of chromosomes, homologous recombination and endoplasmic reticulum stress response were predicted to increase. On the other hand, formation of cytoplasmic aggregates, organismal death, morbidity or mortality, and modification of peptides were predicted to decrease (Supplementary Table 2). Moreover, the top five networks, which exhibited IPA scores ranging from 33 to 39, are listed in Supplementary Table 3. The highest-scoring network was involved in DNA replication, recombination and repair, gene expression, and cell cycle (Fig. 3b). The most significantly affected canonical pathway was determined to be mTOR signalling (Fig. 3c). From the upstream regulation analysis, IPMK and HSPA5 were predicted to be activated, but CD42, MAP4K4, FSH and ERN1 were predicted to be inhibited (Fig. 3d).

**Figure 4:**
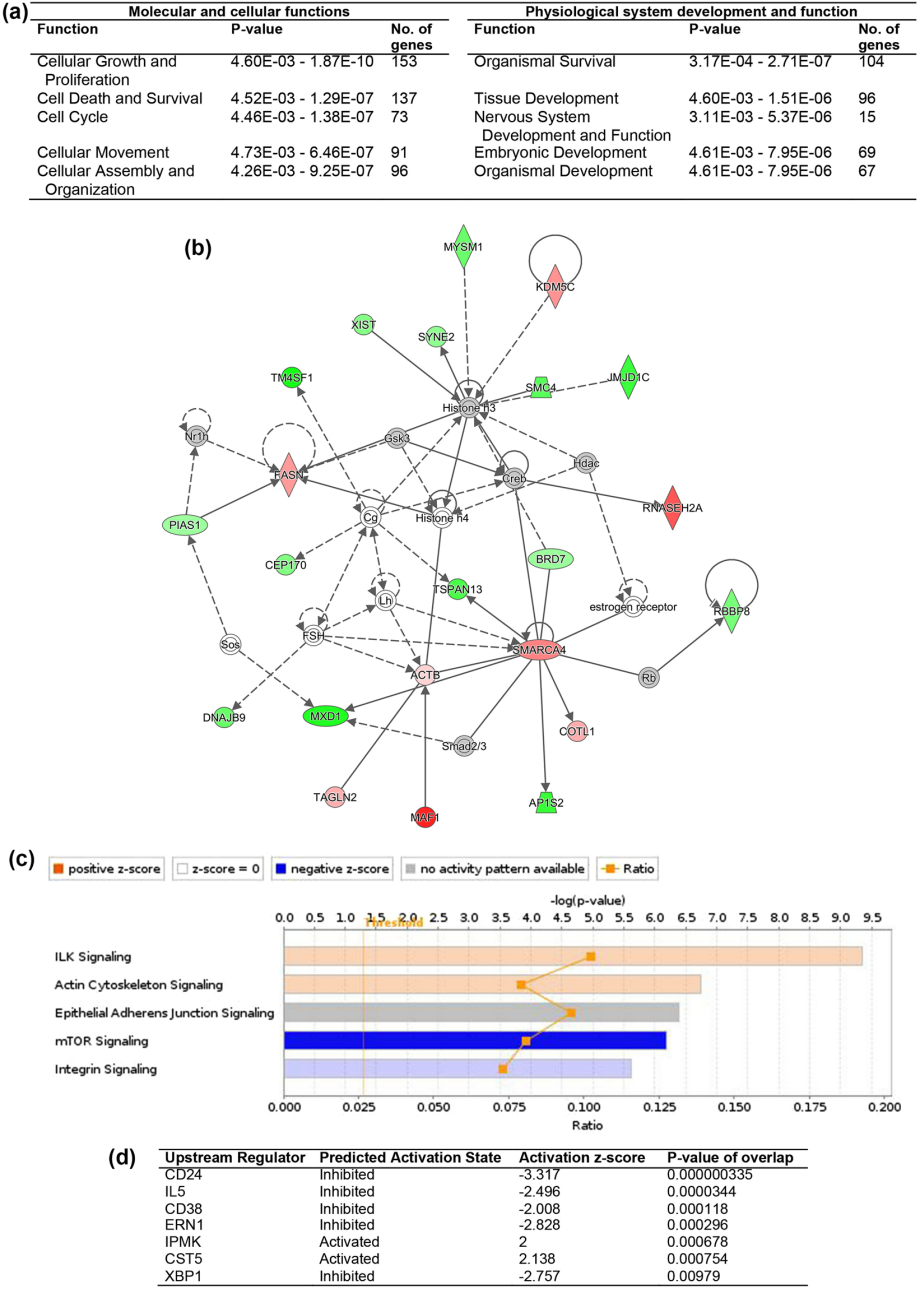
IPA analysis of DEGs from the comparison between miNT blastocysts derived from depleted cells cultured in the presence of TSA and SCNT blastocysts derived from depleted cells cultured in the presence of TSA (n = 3 to 4/group). (**a**) List of the top five biological functions. (**b**) Gene interaction network involved in cellular development, cellular growth and proliferation, and embryonic development. Upregulated and downregulated genes are represented in red and green, respectively. The intensity of colour represents the degree of regulation. Colourless or grey nodes are genes involved in the network which were obtained from the Ingenuity Knowledge Base. The solid and dashed lines represent direct and indirect interactions, respectively. (**c**) The top five statistically significant canonical pathways. (**d**) List of upstream regulators.

For the comparison between miNT and SCNT blastocysts derived from depleted cells cultured in the presence of TSA, 136 genes were upregulated and 262 were downregulated (FDR < 0.05, Supplementary File 3). The top ten most significant DEGs are listed in Supplementary Table 1. From IPA downstream effects analysis, the top significantly affected functions were cellular growth and proliferation, and organismal survival (Fig. 4a). Apoptosis and stress response of cells were predicted to increase, whilst cytokinesis and transcription of DNA were predicted to decrease (Supplementary Table 2). The top five networks, which had IPA scores ranging from 29 to 41, are listed in Supplementary Table 3. The network involved in cellular development, cellular growth and proliferation, and embryonic development consisted of 22 DEGs with an IPA score of 29 (Fig. 4b). The most significantly affected canonical pathway was determined to be ILK signalling (Fig. 4c). Using analysis of upstream regulation, CST5 and IPMK were predicted to be activated and CD24, IL5, CD38, ERN1 and XBP1 to be inhibited (Fig. 4d).

The Venn diagram in Supplementary Fig. 1a shows the number of overlapping DEGs from the three comparison groups. There were 158 common DEGs, which consisted of 14 regulators of transcription (Supplementary File 4). Notably, *ATP5B* and *ATP1A1*, which are involved in OXPHOS, were upregulated in miNT-derived embryos. Using PANTHER, the most affected biological processes were cellular and metabolic processes (Supplementary Fig. 1b). Gene lists for each biological process are shown in Supplementary Table 4.

**Figure 5:**
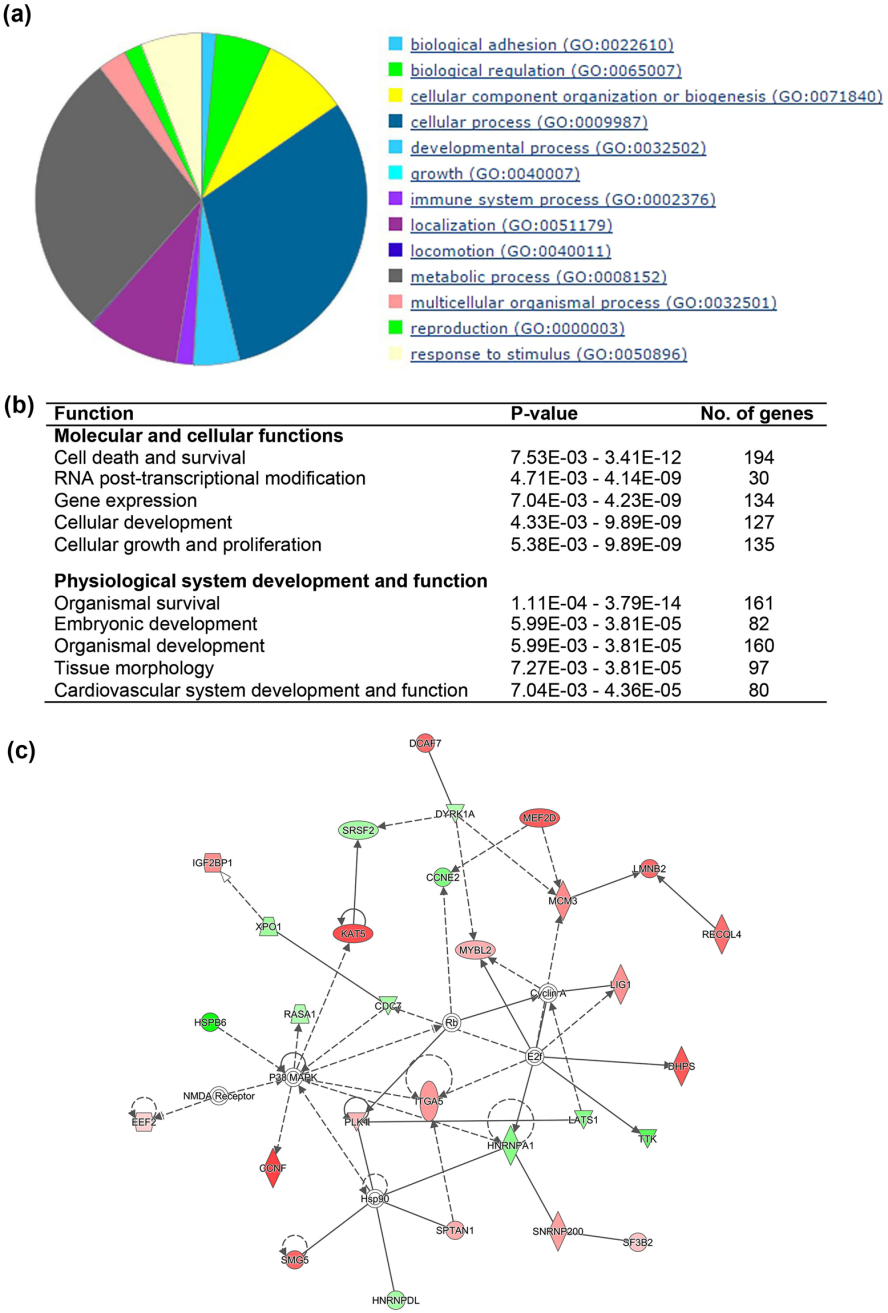
Effects of mtDNA supplementation alone on gene expression of miNT embryos when compared with SCNT blastocysts derived from depleted cells cultured in the presence of TSA (n = 3 to 4/group). (**a**) PANTHER biological process classification. (**b**) List of the top five biological functions. (**c**) Gene interaction network involved in cell cycle, cell death and survival, and DNA replication, recombination, and repair. Upregulated and downregulated genes are represented in red and green, respectively. The intensity of colour represents the degree of regulation. Colourless or grey nodes are genes involved in the network which were obtained from the Ingenuity Knowledge Base. The solid and dashed lines represent direct and indirect interactions, respectively.

Furthermore, we determined the effects of mtDNA supplementation alone and with TSA on the gene expression profiles of miNT-derived embryos when compared with SCNT blastocysts derived from depleted cells cultured in the presence of TSA. There were 348 common DEGs between these two comparisons (Supplementary Fig. 2). The 546 unique DEGs represent the effect of additional mtDNA alone when compared with SCNT blastocysts derived from depleted cells cultured in the presence of TSA. Using PANTHER, the top affected biological processes were determined to be cellular and metabolic processes (Fig. 5a). Genes involved in each biological process have been listed in Supplementary Table 5. By performing IPA, we determined that the most affected molecular and cellular function from the addition of mtDNA alone was cell death and survival, whilst top of the physiological system, development and function classification was organismal survival (Fig. 5b). Moreover, death of embryos, morbidity or mortality, organismal death, expression and translation of mRNA, and modification and metabolism of peptides were predicted to decrease. However, DNA recombination, glycolysis of cells, and organization of the cytoskeleton and cytoplasm were predicted to increase (Supplementary Table 6). The top network function was cell cycle, cell death and survival, and DNA replication, recombination, and repair (IPA score = 39, Fig. 5c).

**Figure 6:**
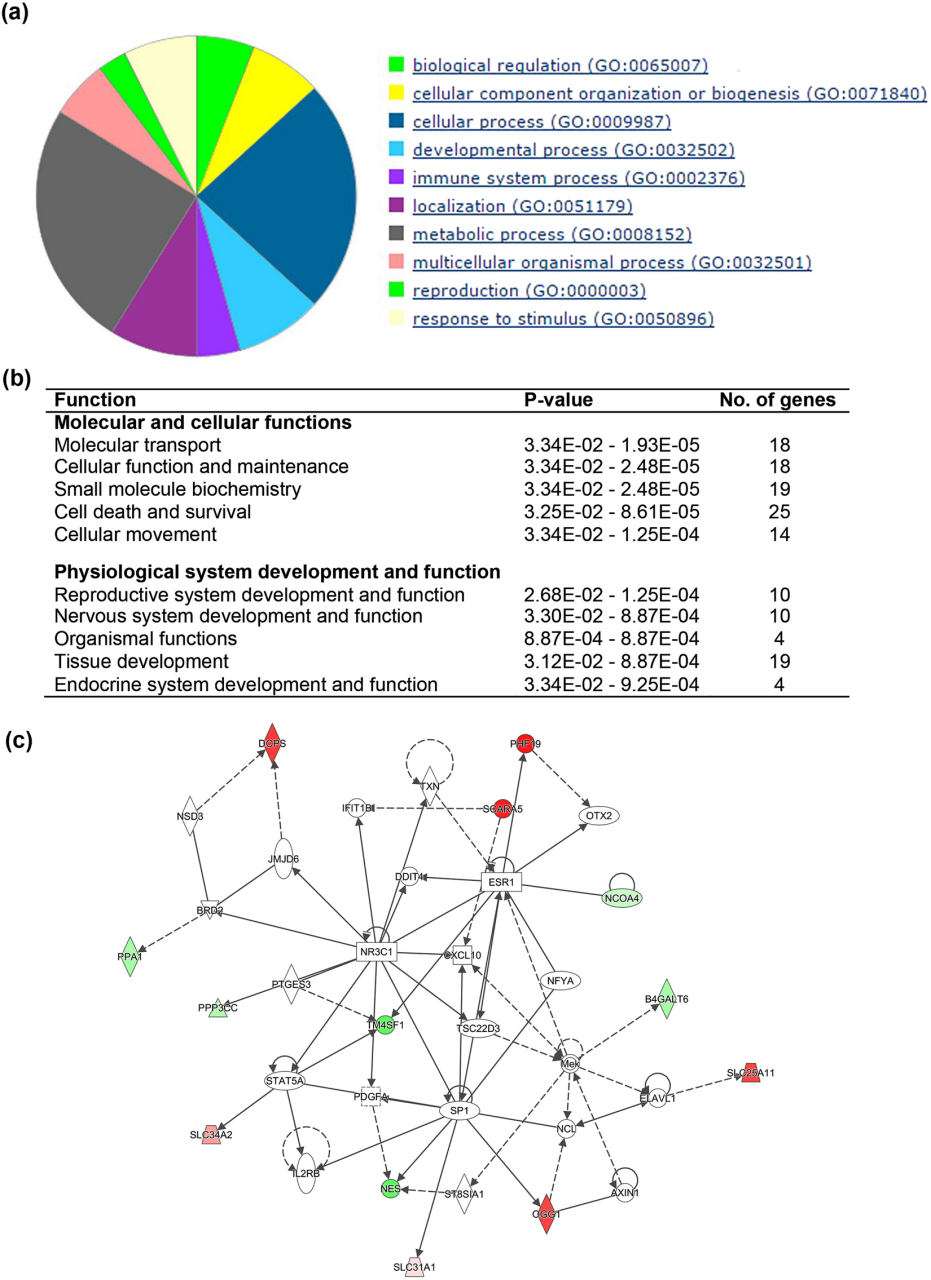
Effects of mtDNA supplementation and TSA on gene expression of miNT embryos when compared with SCNT blastocysts derived from depleted cells cultured in the presence of TSA (n = 3 to 4/group). (**a**) PANTHER biological process classification. (**b**) List of the top five biological functions. (**c**) Gene interaction network involved in gene expression and embryonic development functions. Upregulated and downregulated genes are represented in red and green, respectively. The intensity of colour represents the degree of regulation. Colourless or grey nodes are genes involved in the network which were obtained from the Ingenuity Knowledge Base. The solid and dashed lines represent direct and indirect interactions, respectively.

Focusing on the effects of mtDNA supplementation and TSA when compared with SCNT blastocysts derived from depleted cells cultured in the presence of TSA, we found 50 unique DEGs. The most affected PANTHER biological process was metabolic processes followed by cellular processes (Fig. 6a). Genes involved in each biological process have been listed in Supplementary Table 7. From the IPA results, the most affected molecular and cellular function was molecular transport and, for physiological system, development and function, it was reproductive system development and function (Fig. 6b). The top network with an IPA score of 25 was involved in gene expression and embryonic development functions (Fig. 6c). The biological functions affected by additional mtDNA with and without TSA have been listed in Table 2.

**Table 2:** List of biological functions affected by additional mtDNA with or without TSA when compared with SCNT blastocysts derived from depleted cells in the presence of TSA.

Functions	Additional mtDNA alone	Additional mtDNA + TSA
Glycolysis of cells	↑	N/A
Embryonic death, mortality	↓	↑
DNA repair, cellular function and maintenance	↑	↑
Gene expression	↓	↓
Cell cycle, cell morphology	↑	↓
Cellular movement	↓	↓

We also identified the DEGs that existed between miNT blastocysts derived from depleted cells and nondepleted cells. Only 2 DEGs, *TMEM219* and *APLP1*, were found (FDR < 0.05, Supplementary Table 8), which were upregulated in miNT blastocysts derived from depleted cells. Interestingly, no significant DEGs were identified between miNT blastocysts derived from depleted cells cultured in the presence of TSA and either miNT blastocysts derived from nondepleted or depleted cells cultured in the absence of TSA.

Taken together, depleting mtDNA from donor cells results in upregulation of 2 DEGs in miNT-derived embryos. However, the use of TSA did not have beneficial effects on miNT-derived embryos generated from depleted cells. Notably, miNT-derived blastocysts exhibited very different gene expression profiles compared to SCNT-derived blastocysts. Addition of mtDNA altered gene expression patterns of embryos resulting in decreased cell viability, DNA replication, protein synthesis and post translational modification, but increased expression levels of genes involved in cell cycle and DNA repair when compared with SCNT-derived embryos. However, when compared with SCNT-derived embryos produced from depleted cells cultured in the presence of TSA, mtDNA supplementation alone had beneficial effects in term of decreasing expression of genes associated with embryo death and increasing levels of glycolysis. However, these beneficial effects were not found when mitochondrial supplementation was combined with TSA.

## Discussion

We have used SCNT as a model to determine how additional copies of mtDNA alone can influence the interactions between the nucleus and the mitochondrial genome in the developing embryo. To this extent, we have shown that additional copies of mtDNA can influence developmental rates, blastocyst cell numbers, mtDNA copy number as well as the gene expression profiles of cattle embryos. To our knowledge, this is the first report on how additional copies of mtDNA can influence embryonic gene expression using SCNT as a model.

There are a number of reports showing that embryos produced by SCNT harboured both donor cell and oocyte mtDNA (reviewed in^6^). The mixing of two populations of mtDNA can have serious consequences for embryo development and the health and well-being of intraspecies offspring^19,51,52^. Moreover, aged and damaged mitochondria from somatic cells^53,54^ might be passed onto the embryo and cause disruption to embryo development. Our previous report showed that depleting mtDNA from donor cells prior to SCNT can prevent the transmission of donor cell mtDNA to the embryo as these embryos harboured only oocyte mtDNA. Furthermore, the gene expression patterns of embryos produced from depleted cells were different to those of nondepleted cells^35^. Collectively, this resulted in an improvement to SCNT. However, we acknowledge that the preservation of donor cell mtDNA could be beneficial for interspecies SCNT. It would enable the nucleus to selectively replicate its own mtDNA, and, thus, provide more compatible populations of mtDNA than present in the oocyte of another species.

Nuclear and cytoplasmic maturation are essential processes to developmental outcome^55^. In our work here, we have employed BCB^+^ oocytes, which are indicative of oocytes having achieved developmental competence, namely the nuclear genome having reached its mature state along with sufficient copies of the mitochondrial genome being present^12,56^. We have introduced mtDNA (approximately 560 copies) extracted from BCB^+^ *in vitro* matured oocytes into an enucleated oocyte prior to embryo production to determine how additional mtDNA interacts with the existing mitochondrial and nuclear genomes and the effects that this has on embryo development.

When focussing on miNT-derived embryos cultured in the absence of TSA, we found that blastocyst formation rates for miNT-derived embryos generated from depleted cells were lower than for miNT-derived embryos generated from nondepleted cells, as was the case for SCNT-derived embryos. This likely arises from the adaptation that is required shortly after reconstruction and the potential resetting of the embryonic genome^11^, which enables oocytes of the highest quality to succeed, and suggests that fewer poor quality oocytes are likely to develop to the blastocyst stage, hence the lower blastocyst rates. However, depleting mtDNA from the donor cells positively modulated expression levels of *TMEM219* and *APLP1* in miNT-derived embryos which are involved in embryonic development, most likely as a consequence of the investment in additional mtDNA^11^. Importantly, *TMEM219* has been found to be highly expressed in the inner cell mass of *in vitro* fertilised-derived cattle embryos^57^. Furthermore, a previous report has shown that *APLP1* knockout mice had a reduction in body weight of ~10% when compared with wild type mice^58^. Moreover, there were no differences in mtDNA copy number and the number of cells between miNT-derived blastocysts generated from depleted and nondepleted cells. Notably, the use of TSA did not have a beneficial effect on blastocyst formation rates and did not change gene expression patterns of miNT-derived embryos.

To determine the effects of additional mtDNA, we have compared miNT-derived embryos with SCNT-derived embryos, which were both cultured in the absence of TSA. We found that additional mtDNA did not have a negative effect on embryo development rates as miNT-derived embryos had similar potential to develop to the blastocyst stage when compared with SCNT-derived embryos. Previous reports have shown that mitochondrial supplementation in oocytes increased blastocyst rates as well as improving the quality of embryos in human^59^, cattle^24^ and pigs^11^ when their oocytes were deficient in mtDNA. However, when mtDNA was added to porcine oocytes with sufficient mtDNA, there were no additional benefits^11^. Whilst it has been also reported that the transfer of donor ooplasm containing mtDNA was neither detrimental or beneficial to embryos up to the 8–16 cell stage^60^, donor mtDNA can modify the germline as it is detectable in the offspring’s oocytes^22^. Nevertheless, donor mtDNA appears to decrease in foetal tissues suggesting a skewed bias towards the germline^22^. However, when interspecies SCNT was performed in combination with interspecies ooplasmic transfer, the divergent mtDNA appeared to result in a developmental block at the time of embryonic genome activation^61^, suggesting a clear impact on developmental outcome.

In our study, although miNT-derived embryos had lower cell numbers in blastocysts, their mtDNA copy number was 1.5 to 2 times higher than that of SCNT blastocysts, which suggests that there was effective communication between the nuclear and mitochondrial genomes. The only difference between these two groups was the additional investment of ~ 560 molecules of mtDNA when the embryos were generated. Moreover, miNT-derived embryos cultured in the absence of TSA had very different gene expression profiles when compared with those of SCNT-derived embryos cultured in the absence of TSA. Using IPA to predict the affected functions and networks, additional mtDNA altered gene expression patterns of embryos resulting in cell cycle, cell morphology and DNA repair functions being predicted to increase, whilst cell viability, DNA replication and translation were predicted to decrease when compared with SCNT-derived embryos.

Our previous work showed that TSA positively modulated blastocyst rates and expression levels of genes involved in the embryonic development of SCNT-derived embryos produced from depleted cells^35^. Therefore, SCNT-derived embryos produced from depleted cells and cultured in the presence of TSA were used to determine whether embryos produced by additional mtDNA alone or with TSA had positive effects in terms of gene expression profiles. Gene expression profiles of miNT-derived embryos produced from depleted cells cultured in the presence of TSA (398 DEGs) were deemed to be closer to those of SCNT-derived embryos produced by depleted cells cultured in the presence of TSA than that of miNT-derived embryos produced from depleted cells cultured in the absence of TSA (894 DEGs). However, there were two key positive outcomes from the latter comparison. The levels of expression of genes involved in embryonic death were decreased, whilst expression levels of genes involved in glycolysis were increased in miNT-derived embryos generated by depleted cells in the absence of TSA. On the other hand, the addition of TSA to miNT-derived embryos generated by depleted cells had negative effects as apoptosis was predicted to increase. Moreover, transcription of DNA and cytokinesis were predicted to decrease when compared with SCNT-derived embryos produced by depleted cells in the presence of TSA. These outcomes would suggest that miNT-derived embryos cultured in the absence of TSA were in a state of readiness to undergo cellular proliferation once implantation had taken place as opposed to already undergoing that process as was the case for SCNT-derived blastocysts. Indeed, this may be indicative of a slight delay in embryonic development due to the resetting of the embryonic genome following mtDNA supplementation.

Notably, the levels of expression of *ATP5B* (ATP synthase, H+ transporting, mitochondrial F1 complex, beta polypeptide) and *ATP1A1* (ATPase Na+/K+ transporting subunit alpha 1) were upregulated in miNT-derived embryos when compared with SCNT-derived embryos, irrespective of whether TSA was used. The nuclear-encoded *ATP5B* is involved in OXPHOS which encodes a subunit of the mitochondrial ATP synthase^62^. This outcome correlated with the higher mtDNA copy number in miNT-derived embryos. *ATP1A1* is one of the most important genes involved in embryonic development as *ATP1A1* knockout mouse embryos are unable to form blastocysts *in vitro*. Although these embryos can develop to blastocyst *in vivo*, no offspring have been derived from *ATP1A1* knockout mice^63^.

Taken together, by adding additional copies of the mitochondrial genome to a developmentally competent oocyte and replacing the chromosomes with donor cells harbouring the same chromosomal DNA complement, we have observed the degree to which the mitochondrial genome alone can influence cellular and developmental outcome. Furthermore, our data show that the use of additional copies of mtDNA negated the impact that a reprograming agent can have on development^64^, as TSA was not able to affect global gene expression patterns further. Indeed, we have previously observed that modulation of mtDNA copy number can promote differentiation in tumour cells that fail to undergo differentiation^65^, again highlighting the importance of the synergistic relationship between the chromosomal and mitochondrial genomes to mediate developmental outcomes.

During oogenesis, the synergy between the nuclear and mitochondrial genomes is an important determiner of how the resultant embryo will perform. However, it is clearly evident that additional copies of mtDNA introduced into either BCB^+^ or BCB^−^ oocytes at the time of fertilisation^11^ or as SCNT is performed can readdress the balance that has already been achieved during oogenesis and would further explain why mtDNA copy number is strictly regulated during early development^66^. This is perhaps best highlighted by events that take place when the embryo first compartmentalises at the blastocyst stage. At this stage, the trophectodermal cells initiate replication as they undergo differentiation but the naïve pluripotent cells of the inner cell mass, which give rise to the embryo proper and the fetus, do not replicate their mtDNA and continue to reduce numbers with each stage of division. This enables naïve cells to establish the mtDNA set point, which determines whether these cells can initiate and complete differentiation^13,67–70^.

In conclusion, we have shown, in a model where the genetics of the nuclear and mitochondrial genomes are highly regulated, the impact that additional copies of mtDNA can have on embryo development. Whilst miNT-derived embryos had higher mtDNA copy number at the blastocyst stage and had different gene expression profiles when compared with SCNT-derived embryos, they exhibited lower cell numbers at the blastocyst stage. Nevertheless, their gene expression profiles suggested that they were in readiness to promote cellular proliferation. Importantly, our outcomes indicate that a comparatively small amount of additional mtDNA can influence embryo development by potentially mediating the resetting of the embryonic genome. This demonstrates that the nuclear and mitochondrial genomes establish a position of interplay to promote embryonic development and that there may be beneficial effects of additional mtDNA over the use of TSA in terms of regulating gene expression in SCNT-derived embryos.

## Methods

All chemicals and reagents were purchased from Sigma-Aldrich (St. Louis, MO, USA), unless otherwise specified.

### Ethics approval and consent

Animal ethics committee approvals were not required as cattle samples were obtained from animals that were slaughtered for commercial food production. None of the authors were involved in the decision to slaughter any animals used in this study. Experimental procedures were conducted in accordance with the Australian Code for the Care and Use of Animals for Scientific Purposes, 8th Edition 2013.

### Donor cell preparation

Donor cells were prepared, as previously described^35^. Briefly, mtDNA was depleted from skin fibroblasts obtained from an adult cow by culturing in media (Dulbecco’s Modified Eagle Medium (DMEM, Gibco, Grand Island, NY, USA) supplemented with 10% fetal bovine serum (FBS, Gibco)) and, additionally, supplemented with 20 µM ddC and 50 µg/ml Uridine at 37 °C under humidified atmosphere of 5% CO_2_ in air for 30 days to generate mtDNA depleted cells. The culture medium was changed every 24 h. Cells were passaged once they reached 80% confluency. Prior to embryo production, mtDNA-depleted cells were harvested using TrypLE^TM^ Express (Gibco) and resuspended in EMCARE™ holding solution (ICPbio Reproduction, Timru, Auckland, New Zealand). mtDNA depleted cells cease to proliferate after extensive depletion and are deemed to be in a G0 state, as previously shown^35,71^. For nondepleted cells, fibroblast cells were synchronised to the G0/G1 phase by culture in DMEM supplemented with 0.5% FBS at 37 °C under humidified atmosphere of 5% CO_2_ in air for 2 to 3 days.

### Oocyte preparation

Cumulus oocyte complexes (COCs) were aspirated from 2-3 mm follicles from abattoir-derived ovaries. COCs were stained with 20 µM BCB in *in vitro* maturation (IVM) media (TCM199 supplemented with 10% FBS, 50 IU/ml hCG ((Chorulon^®^, Intervet, Bendigo East, VIC, Australia)), 0.5 µg/ml FSH ((Folltropin^®^-V, Bioniche Animal Health, Belleville, ON, Canada)), 1 µg/ml 17ß-estradiol) at 38.5 °C under humidified atmosphere of 5% CO_2_ in air for 1 h. Only BCB^+^ COCs were selected and then washed five times before culture in IVM media at 38.5 °C under humidified atmosphere of 5% CO_2_ in air for 19 h. After that, cumulus cells were removed from COCs by repeated pipetting in 0.2% hyaluronidase. The denuded oocytes were then washed five times in EMCARE™ holding solution.

### Mitochondrial isolation

Mitochondria were isolated from pooled IVM oocytes obtained from 10 cows otherwise the protocol described previously^11^ was followed. The denuded oocytes were washed five times in Dulbecco’s phosphate-buffered saline (DPBS, Gibco) supplemented with 0.1% polyvinylpyrrolidone (PVP) and then resuspended in a glass homogeniser containing 5 ml mitochondrial isolation solution (20 mM Hepes pH 7.6, 220 mM Mannitol, 70 mM sucrose, 1 mM EDTA) supplemented with 2 mg/ml bovine serum albumin (BSA). The oocytes were homogenised by 10 strokes with a drill-fitted pestle at 4 °C. The homogenate was centrifuged at 800 g for 10 min at 4 °C and the supernatant was then centrifuged at 10, 500 g for 20 min at 4 °C. The supernatant was removed. The pellet was resuspended in the mitochondrial isolation solution without BSA and centrifuged at 10, 500 g for 20 min at 4 °C. The mitochondrial pellet was concentrated by loading the isolate into a tip sealed-straw (Stripper tips, Origio, Charlottesville, VA, USA) and centrifuged at 10, 000 g for 40 sec at 4 °C.

### Embryo production using mitochondrial supplementation and nuclear transfer (miNT) and Somatic Cell Nuclear Transfer (SCNT)

To determine the effects of additional mtDNA on embryo development, six different groups of embryos were generated and four replicates were generated for each treatment:

1. miNT-derived embryos generated from nondepleted cells in the absence of TSA;
2. SCNT-derived embryos generated from nondepleted cells in the absence of TSA;
3. miNT-derived embryos generated from depleted cells in the absence of TSA;
4. SCNT-derived embryos generated from depleted cells in the absence of TSA;
5. miNT-derived embryos generated from depleted cells in the presence of TSA; and
6. SCNT-derived embryos generated from depleted cells in the presence of TSA.

Enucleation was performed according to our previous report^35^. Briefly, the spindle was aspirated from BCB^+^ metaphase II (MII) oocytes in 5 µg/ml cytochalasin B using a micropipette (OD 12 µm, ID 9 µm, The Pipette Company, Adelaide, SA, Australia) under the Oosight™ Imaging System (CRi, Woburn, MA, USA). Then, enucleated oocytes were separated into two groups for miNT- and SCNT-derived embryo production. For miNT, 1 ηl of mitochondrial isolate was injected into the enucleated oocytes. At the end of each round of injection, several aliquots of 1 ηl of mitochondrial isolate were injected into PCR tubes for subsequent analysis of mtDNA copy number (see: ‘mtDNA copy number analysis’). Then, a single donor cell from the cohort of mtDNA depleted cells (passage number 12) or nondepleted cells (passage number 9) was inserted into the perivitelline space of each enucleated oocyte. The donor cell-cytoplast couplets were fused in Zimmermann fusion media^72^ using 2 direct current pulses of 22 V of 14 µs generated by a BTX Electro Cell Manipulator 200 (BTX, San Diego, CA, USA). The successfully fused oocytes were activated using 7% ethanol for 5 min at room temperature followed by 1.25 µg/ml cytochalasin D and 10 µg/ml cycloheximide at 38.5 °C under humidified atmosphere of 5% CO_2_, 5% O_2_ and 90% N_2_ for 5 h. After activation, the embryos (20 embryos per 100 µl medium) were cultured in CR1aa medium^73^ at 38.5 °C under humidified atmosphere of 5% CO_2_, 5% O_2_ and 90% N_2_ for 7 days. 50 µl of CR1aa medium was replaced at days 3 and 5 of culture. For SCNT, embryo production was the same as for miNT except that mitochondrial supplementation was not performed^35^.

To determine whether the nuclear reprograming reagent Trichostatin A (TSA) enhances miNT- and SCNT-derived embryo production, a few minutes after fusion, the reconstructed oocytes produced from depleted cells were separated into two groups, absence and presence of TSA. For the presence of TSA group, the reconstructed oocytes were placed in EMCARE™ holding solution supplemented with 50 nM TSA for 1 h. Successfully fused oocytes were activated and cultured in medium supplemented with TSA for up to 10 h^44^. After that, embryos were continuously cultured in the medium without TSA for 7 days until they reached the blastocyst stage. 50 µl of CR1aa medium was replaced at days 3 and 5 of culture (presence of TSA group). For the absence of TSA group, the reconstructed oocytes were activated and cultured in the same media as those cultured in the presence of TSA except that TSA was omitted.

For each round of embryo production, PA served as the control group. The MII oocytes were directly activated and cultured in the same media as the miNT and SCNT groups in the absence of TSA.

### Assessment of cell number of miNT- and SCNT-derived embryos

Embryo staining was performed according to our previous report^35^. miNT- and SCNT-derived embryos at the blastocyst stage (3 individual embryos per group taken from different replicates of embryo production) on day 7 of culture were fixed in 4% paraformaldehyde and then permeabilized in 0.1% Triton X-100 and stained with 10 µl/ml of Hoechst 33258. The Hoechst stained nuclei were visualized and counted under UV light using an inverted confocal microscope (FV1200, Olympus, Tokyo, Japan).

### mtDNA copy number analysis

DNA was prepared and mtDNA copy number analysis was performed according to our previous report^35^. Briefly, total DNA was extracted from embryos at the blastocyst stage (10 individual embryos per group) using the QIAamp DNA Micro Kit (Qiagen, Hilden, Germany), according to the manufacturer’s instructions. Primers were designed from the NADH dehydrogenase subunit 2 (*ND2*) region (F: TATACGACTCACGTATTCTACC, R: CTTTGAAGGCTCTTGGTCTG^35^). The quantification of mtDNA was performed by real-time polymerase chain reaction (PCR) using a Rotorgene 3000 real-time machine (Corbett Life Science, Sydney, NSW, Australia). PCR mixtures (final volume of 20 µl) contained 1 × SensiMix^TM^ SYBR Hi-ROX (Bioline), 0.25 µM of each primer and 2 µl of embryo DNA or 2 µl of DPBS containing 1 ηl aliquot of mitochondrial isolate. PCR amplification was carried out at 95 °C for 15 min, followed by 45 cycles of 95 °C for 15 s, 55 °C for 15 s and 72 °C for 15 s. The final elongation step was performed at 72 °C for 15 s. The second extension phase was set at 74 °C for 15 s to exclude the negative effects of primer-dimerisation and the second extension fluorescence signals from the FAM/Sybr channel were acquired.

The *ND2* region was amplified from total DNA of nondepleted cells to generate standards for real-time PCR. The PCR products were purified using the Isolate II PCR Gel Kit (Bioline, Alexandria, NSW, Australia), according to the manufacturer’s instructions. For each run, a standard curve was generated from 10-fold serial dilutions (10^−1^ to 10^−8^). Each sample was analysed in triplicate to obtain the mean number of mtDNA copies.

### RNA sequencing and analysis

RNA extraction from single blastocysts from each group (3 to 4 individual embryos per group) was performed using the Arcturus® PicoPure® RNA Isolation Kit (Life Technologies), according to the manufacturer’s instructions. cDNA synthesis from total RNA for RNA sequencing was performed using the Ovation^®^ RNA-Seq System V2 (NuGen, San Carlos, CA, USA), according to the manufacturer’s instructions. First strand cDNA synthesis was performed using a DNA/RNA chimeric primer mix and reverse transcriptase. Second strand cDNA was synthesised by DNA polymerase. After that, the double stranded cDNA with a unique DNA/RNA heteroduplex was amplified by SPIA^®^ amplification in the presence of RNase H, a DNA/RNA chimeric SPIA^®^ primer, and DNA polymerase. The cDNA was purified using the Agencourt AMPure XP system (Beckman Coulter, Brea, CA, USA) and was then sheared using Covaris sonication (Covaris, Woburn, MA, USA), according to the manufacturer’s instructions. Sequencing library construction was performed using Ovation Ultralow System V2 (NuGen), according to the manufacturer’s instructions. Libraries were quantified using a Qubit fluorometer (ThermoFisher Scientific, Waltham, MA, USA) and an Agilent 2100 Bioanalyzer (Agilent Technologies, Santa Clara, CA, USA), and quantitative real time PCR. 12 pM of the library pool were clustered in two lanes of an Illumina HiSeq 1500 Rapid with 100 bp pair end read lengths using on board clustering, according to the manufacturer’s instructions (Illumina, San Diego, CA, USA) at the Monash Health Translation Precinct Biomedical Genomics Facility. Raw reads from the libraries were quality checked using the FastQC tool (http://www.bioinformatics.bbsrc.ac.uk/projects/fastqc/). Sequence reads were assembled and mapped to the bovine reference genome obtained from the NCBI database (GenBank assembly accession: GCA_000003055.4; NCBI Genome information: NCBI genome/82 (*Bos taurus*)) using the TopHat aligner version 2.0.14 (https://ccb.jhu.edu/software/tophat/manual.shtml). The reads mapped to each gene were counted using the featureCounts tool^74^. Differential expression analysis was performed using the edgeR package^75^ by pairwise comparisons between experimental groups. Multiple testing correction was performed using edgeR (empirical Bayes estimation) by applying the Benjamini and Hochberg method^76^ to p-values, to control for false-discovery rate (FDR). For each comparison, genes with FDR < 0.05 were considered as differentially expressed^35,77,78^. Pathway analysis was performed using Ingenuity Pathway Analysis (IPA, QIAGEN Redwood City, www.qiagen.com/ingenuity), as described in^11^. P-values were calculated by Fishers Exact Test. Moreover, gene IDs for DEGs were uploaded into PANTHER (http://pantherdb.org/index.jsp) for biological process classification analysis^79^.

### Statistical analysis

Data for embryo development and cell number were analysed by ANOVA and means were compared using Duncan’s Multiple Range Test using Statistical Analysis System version 9 (SAS Inst. Inc., Cary, NC, USA). Statistical analysis of mtDNA copy number data was evaluated by two-way ANOVA with Sidak’s multiple comparisons test using GraphPad Prism version 6.01 (GraphPad Software Inc., La Jolla, CA, USA). The number of differentially expressed genes was analysed using edgeR at FDR < 0.05.

### Data availability

RNA-sequencing data for miNT and SCNT embryos are deposited at the Sequence Read Archive with SRA accession numbers: SRP098838 and SRP102750, respectively.

## Electronic supplementary material

Supplementary Information

Supplementary File 1

Supplementary File 2

Supplementary File 3

Supplementary File 4
