# Supplementary material for: Additional mitochondrial DNA influences the interactions between the nuclear and mitochondrial genomes in a bovine embryo model of nuclear transfer
Source: Sci Rep. 2018 May 8;8:7246. doi: 10.1038/s41598-018-25516-3 (PMC5940817; doi:10.1038/s41598-018-25516-3)
Supplement: Supplementary file 1 — Supplementary Information [file 41598_2018_25516_MOESM1_ESM.pdf]

**Supplementary Information**

# Additional mitochondrial DNA influences the interactions between the nuclear and mitochondrial genomes in a bovine embryo model of nuclear transfer.

Kanokwan Srirattana[1,2] and Justin C. St. John[1,2*]

[1] Centre for Genetic Diseases, Hudson Institute of Medical Research, Clayton, VIC 3168, Australia.

[2] Department of Molecular and Translational Sciences, Monash University, Clayton, VIC 3168, Australia.

[*]Corresponding author

E-mail: justin.stjohn@hudson.org.au

**Supplementary Table 1.** List of the top ten most significant differentially expressed genes.

| Top ten DEGs | Fold change | Regulation | FDR |
|---|---|---|---|
| ***Comparison between miNT and SCNT blastocysts derived from depleted cells*** | | | |
| *XIST* | 4.910942736 | Down | 0.0044 |
| *TMEM41B* | 7.140375772 | Down | 0.0105 |
| *RPL23A* | 20.11221399 | Down | 0.0105 |
| *RPL17* | 6.502524816 | Down | 0.0105 |
| *BSG* | 5.555820543 | Up | 0.0105 |
| *ARF4* | 5.176582618 | Down | 0.0105 |
| *CEP135* | 5.582843056 | Down | 0.0108 |
| *TPT1* | 9.272767615 | Down | 0.0109 |
| *ZDHHC6* | 4.688089135 | Down | 0.0109 |
| *RAP1A* | 7.490177979 | Down | 0.0109 |
| ***Comparison between miNT blastocysts derived from depleted cells cultured in the absence of TSA and SCNT blastocysts derived from depleted cells cultured in the presence of TSA*** | | | |
| *PNRC2* | 6.759891075 | Down | 0.00265 |
| *BSG* | 6.639156685 | Up | 0.00265 |
| *XIST* | 4.462957289 | Down | 0.00265 |
| *AMD1* | 7.356400369 | Down | 0.00265 |
| *PAPOLA* | 4.011105744 | Down | 0.00265 |
| *TMEM41B* | 6.89714836 | Down | 0.00265 |
| *CEP135* | 6.130479841 | Down | 0.00265 |
| *RPL23A* | 21.30322581 | Down | 0.00265 |
| *FLNB* | 6.802193281 | Up | 0.00374 |
| *LARP4* | 4.453686473 | Down | 0.00374 |
| ***Comparison between miNT blastocysts derived from depleted cells cultured in the presence of TSA and SCNT blastocysts derived from depleted cells cultured in the presence of TSA*** | | | |
| *REEP3* | 7.851672511 | Down | 0.00285 |
| *RPL23A* | 11.6237204 | Down | 0.00285 |
| *TMEM41B* | 5.073565597 | Down | 0.00531 |
| *SSB* | 5.296355642 | Down | 0.00531 |
| *MOSPD1* | 5.256126509 | Down | 0.00531 |
| *RAN* | 10.68118643 | Down | 0.00531 |
| *SDCBP* | 3.858392738 | Down | 0.00642 |
| *JMJD1C* | 5.344296314 | Down | 0.00642 |
| *LOC782101* | 5.084126757 | Down | 0.00642 |
| *NEMF* | 4.355988061 | Down | 0.00649 |

**Supplementary Table 2.** List of the biological functions.

| Categories | Functions annotation | p-Value | Predicted State | Activation z-score | No. of genes |
|---|---|---|---|---|---|
| ***Comparison between miNT and SCNT blastocysts derived from depleted cells*** | | | | | |
| Cell Cycle, Cell Morphology | formation of spindle apparatus | 0.000662 | Increased | 2 | 8 |
| | formation of mitotic spindle | 0.00171 | Increased | 2 | 7 |
| Cell Cycle, DNA Replication, Recombination, and Repair | recombination of cells | 0.000000203 | Increased | 2.514 | 14 |
| | homologous recombination of cells | 0.00000047 | Increased | 2.514 | 13 |
| | homologous recombination | 0.000000716 | Increased | 2.177 | 15 |
| Organismal Survival | organismal death | 3.03E-11 | Increased | 2.177 | 140 |
| Cell Death and Survival | cell viability | 0.000000326 | Decreased | -2.295 | 74 |
| | cell survival | 0.000000407 | Decreased | -2.206 | 76 |
| Cellular Movement | cell movement | 0.00003 | Decreased | -2.136 | 101 |
| DNA Replication, Recombination, and Repair | metabolism of DNA | 0.00000529 | Decreased | -2.426 | 28 |
| | DNA replication | 0.0000418 | Decreased | -2.062 | 19 |
| Post-Translational Modification | ubiquitination of protein | 0.0000377 | Decreased | -2.164 | 23 |
| Protein Synthesis | translation | 0.0000873 | Decreased | -2.085 | 18 |
| ***Comparison between miNT blastocysts derived from depleted cells cultured in the absence of TSA and SCNT blastocysts derived from depleted cells cultured in the presence of TSA*** | | | | | |
| Cell Cycle, Cellular Assembly and Organization, DNA Replication, Recombination, and Repair | segregation of chromosomes | 0.000335 | Increased | 2.218 | 15 |

| | | | | | |
|---|---|---|---|---|---|
| Cell Cycle, DNA Replication, Recombination, and Repair | homologous recombination | 0.000426 | Increased | 2.177 | 15 |
| | recombination of cells | 0.000109 | Increased | 2.514 | 14 |
| | homologous recombination of cells | 0.000165 | Increased | 2.514 | 13 |
| Cellular Compromise, Cellular Function and Maintenance | endoplasmic reticulum stress response | 0.00157 | Increased | 2.213 | 16 |
| Cellular Assembly and Organization | formation of cytoplasmic aggregates | 0.000097 | Decreased | -2.578 | 8 |
| Organismal Survival | organismal death | 1.36E-18 | Decreased | -2.187 | 243 |
| | morbidity or mortality | 3.88E-18 | Decreased | -2.158 | 244 |
| Protein Synthesis | modification of peptide | 0.0000965 | Decreased | -2.236 | 15 |
| ***miNT blastocysts derived from depleted cells cultured in the presence of TSA and SCNT blastocysts derived from depleted cells cultured in the presence of TSA*** | | | | | |
| Cell Death and Survival | apoptosis | 0.00000172 | Increased | 2.298 | 101 |
| Cellular Compromise | stress response of cells | 0.00235 | Increased | 2.63 | 8 |
| Cellular Compromise, Cellular Function and Maintenance | endoplasmic reticulum stress response | 0.000266 | Increased | 2.19 | 11 |
| | endoplasmic reticulum stress response of cells | 0.00129 | Increased | 2.219 | 6 |
| Cell Cycle | M phase | 0.000475 | Decreased | -2.189 | 14 |
| Cell Cycle, Cellular Movement | Cytokinesis | 0.00148 | Decreased | -2.189 | 11 |
| Cell Morphology | orientation of cells | 0.000019 | Decreased | -2.121 | 13 |
| Cellular Movement | migration of cells | 0.00356 | Decreased | -2.018 | 64 |
| Gene Expression | transcription of DNA | 0.000297 | Decreased | -2.188 | 60 |

**Supplementary Table 3.** List of the top five networks generated using IPA.

| **Genes in network (Focus gene in bold)** | **IPA score** | **No. of focus genes** | **Top functions** |
|---|---|---|---|
| **Comparison between miNT and SCNT blastocysts derived from depleted cells** | | | |
| **AKT1S1, ATP1A1, ATP5B, ATP6AP2, CALB1, CALM1 (includes others), CAPZA2, FKBP1A, HIF3A, HMGN1,** Insulin, **MLST8**, MTORC1, **NDUFA10**, NFkB (complex), **NOP58, NUCB2,** p70 S6k, **PDIA3**, PP2A, **PSMD12, RAP1GDS1, RHOC, RICTOR, RPL17, RPS6, RRAS2, RUVBL2,** SRC (family), **SUMO1, TAX1BP1, TFG, TPT1, UQCRB, WDR34** | 40 | 29 | Cancer, Organismal Injury and Abnormalities, Respiratory Disease |
| **ACTB, BSG, DDX5, DSC2, DSG2,** ERK, **HNRNPA1, HNRNPDL,** Hsp90, **HTATSF1, IGF2BP1, IPMK, JUP, KLF5, MET, MIF, MYL9, MYO1C, NPM1,** p85 (pik3r), **PLK1, PSMA3**, Rb, **RBBP8,** RNA polymerase II, Rnr, **RPS2, RPS3, RPS15A, SMAD5, SMG5, SNRNP200, TAGLN2, THOC1, UACA** | 40 | 29 | Cellular Development, Cellular Growth and Proliferation, Cardiac Arrythmia |
| 26s Proteasome, Alp, **ATP6V1A, ATXN2L, BCL2L1, CBX3, CD47, CTSD, DBN1, DDB1, DDX6, DDX28**, Eif4g, **GALNT3, HEXA, HNRNPA2B1**, Hsp70, IL12 (family), **INTS3, MAPK8, MFN1, MOV10, NCOA1,** P-TEFb, **PMS1, POLR2A, PRDX3, PTOV1, RBBP6, RIMKLB, SUPT5H, SUPT6H, TFEB, TOB1, UPF3B** | 40 | 29 | Gene Expression, Cell Morphology, Cellular Function and Maintenance |
| **ACTN1, ACTR2, ADAM9,** Alpha tubulin, **CCDC22, CD2AP, CEP290, CKB, CLIC4, CLIP1, DGKZ,** Dynein, **DYNLT3**, ERK1/2, **FAM120A, FUT8**, Importin beta, **MADD**, MAP2K1/2, **MKS1**, NADPH oxidase, **NUMA1**, **PIK3CA, OLDIP3, PRDX2, RAB8B, RAD50, RAN, RAP1A, RPL23A, RPS6KB1, SMC3**, SYK/ZAP, **WASHC1, YES1** | 38 | 28 | Cellular Assembly and Organization, Cellular Function and Maintenance, Cell Morphology |
| Akt, **CEBPZ, CHORDC1,** Cofilin, **DNM2, ABP5, GNAI3, GNB4, HYOU1**, LDL, Mek, Mlc, **MYH9, NDUFV1, NRAS, PDCD10, PIP5K1C, PPP1R12A, PPP1R14A,** Ppp2c, **PPP2CA, PPP2R5D**, PRKAA, **RABGGTB**, Rock, **ROCK2, SDCBP, EC24A, SGO2, SMPD1, TFAM, TKT, UBA6, UBE3A, USP8** | 36 | 27 | Cell-To-Cell Signaling and Interaction, Cellular Compromise, Cell Signaling |

| | | | |
|---|---|---|---|
| **miNT blastocysts derived from depleted cells cultured in the absence of TSA and SCNT blastocysts derived from depleted cells cultured in the presence of TSA** | | | |
| **ACTR2, ACTR3, ATN1, BAZ2A, BEND3, CAMSAP3, DMAP1, ELL2, H3F3A/H3F3B,** Histone h3, **HTATSF1, IFITM3, JMJD1C, MGLL, MYSM1, NCAPD2, NMD3**, P-TEFb, **PHC1, PRRC2A, RAN, RAVER1,** RNA polymerase II, **RPL18, SF3A2, SF3B2, SH3GL1, SMC4, SNRPB2, SUPT5H, SUPT6H, SYNE2, THOC1, USP3, XIST** | 39 | 32 | DNA Replication, Recombination, and Repair, Gene Expression, Cell Cycle |
| **ACO2, ALDOA, APH1A, APP, ARF4, ATP5B, ATP5D, ATP6V1A, BIN1, CDK5RAP2, CFL2, CKB, CLIC4, CLTB, CNTNAP1, CTSD, CXXC1,** cytochrome C, **DYNLL2, EEF1G, GNMT, GPANK1, HIST1H2BD, MTHFD1L, MTHFR, NDUFA5,** Notch, **NPC1, SEC23A,** Secretase gamma, **SETD1A, SRFBP1, STX12, VPS29, VPS35** | 39 | 32 | Lipid Metabolism, Molecular Transport, Small Molecule Biochemistry |
| **ADRM1, AP3D1, AP3S1, ATP6AP2, CALM1 (includes others), CAPZA2, CDC42EP1,** Clathrin, **CLCN3, DYNC1H1, ECSIT, HIP1,** IL1, **LUC7L3, MAP3K7, MTDH,** NADPH oxidase, NFkB (complex), **PDCD11, PGD, PIAS4, PICALM, RAP1GDS1, RASSF5, RRAS2, SENP6, SMAD7,** Srebp, **SUMO1, TAB2, TAX1BP1, TRIM32, UBE2D3, WDR34, YES1** | 35 | 30 | Gene Expression, Cell Death and Survival, Decreased Levels of Albumin |
| **ACADVL, ALAS1, AP1S2, CLK1, COTL1, DNMT3B, DPF2, ESRRA, FARP1, FASN, FOXO4, GALT,** Hdac, **IFRD1, LHPP, MBNL1, MCRS1, MXD1,**N-cor, **NAMPT**, Nr1h, **PAF1, PDP1, PIAS1, POU5F1, PPRC1, PRDM14, PRPF6, PRRC2C, SBNO2, SEC62, SLC30A1,** Smad2/3, **SMARCA4**, Sos | 35 | 30 | Cellular Function and Maintenance, Gene Expression, Cellular Development |
| **BAP1, CCNE2, CCNG1, CLN6, DCAF7, DHPS, DYRK1A,** E2f, **ERO1A, FHL3, FURIN,** Gamma tubulin, hemoglobin, **HIF1A,** Hsp70, **HSPA4, LARP7, LMNB2, MCM3, MEF2D, NDRG1, ORC4, POLRMT**, Rb, **RBBP8, RECQL4, SKA2, SLC29A1, SMAD5, SMG5, SRSF2, TCEB1, TUBGCP5,** Ubiquitin, **XPNPEP2** | 33 | 29 | Developmental Disorder, Embryonic Development, Organismal Development |

| Comparison between miNT blastocysts derived from depleted cells cultured in the presence of TSA and SCNT blastocysts derived from depleted cells cultured in the presence of TSA | | | |
|---|---|---|---|
| Actin, Akt, **ANKRD1, CAPZB, DDX5, EIF2A, FHL3, GNAI3, GNB4,** Hsp90, **HTATSF1, KLHL2, LAMP1, MGLL, MYCBP, MYL9, MYO1C, NCL,** Notch, **NPM1, NRAS, OGG1, PIK3R2, PIP5K1C,** PP1 protein complex group, RNA polymerase II, Rnr, **RPS3, RPS6, RPS15A, S100A11, SDCBP, SRFBP1, SYCP3, TES** | 41 | 28 | Cancer, Hematological Disease, Organismal Injury and Abnormalities |
| **ACTN4, B4GALT6,** Cofilin, **DHFR, DMAP1, FABP5, HIP1, KLHL20,** LDL, **LUC7L3**, Mek, Mlc, **MPRIP, MTDH,** MTORC1, **NAMPT, NCOA4**, NFkB (complex), **NLRX1, PDCD11,** PP2A, **PPP1R12A, PRKAA1, PTPRA,** Rac, **RHOA, RND3, ROCK2, RRAS2, SLC7A5, SND1, SUMO1, SWAP70, TAX1BP1, YES1** | 39 | 27 | Cellular Growth and Proliferation, Cell-To-Cell Signaling and Interaction, Cellular Assembly and Organization |
| **ACTN1, ARRB2, ATP1A1, CD9, CD2AP, CHORDC1, DNM2,** Dynein, **DYNLT3, ELF1,** ERK1/2, Erm, **FKBP1A, GLIPR2, HLTF,** Integrin, **MOB4, MYH9, NBN,** p70 S6k, **PDCD10,** Ppp2c, **PTP4A2, PTPN12, RAD50, RAP1A,** Rock, RPA, **SLC39A10,** SRC (family), **TAPBP, THBD, TPR, TPT1, VCL** | 37 | 26 | Cell-To-Cell Signaling and Interaction, Cell-mediated Immune Response, Cellular Development |
| 26s Proteasome, **ACADVL**, Alpha tubulin, AMPK, **ATP5B, ATP6V0D2, BCLAF1, BSG, CDK13, CHD1, CKB, CLIP1,** Cyclin E, **DNMT3B, DYNC1H1, ELL2,** ERK, **ERO1A, ESRRA, HIF1A, JAKMIP2**, mediator, **MGAT1**, Mitochondrial complex 1, **MTHFD1L, PDP1, PRRC2C, RTN4, SIRT1, STUB1,** TCR, **TOP1, UBR4, UCHL3,** Vegf | 37 | 26 | Cell-To-Cell Signaling and Interaction, Hematological System Development and Function, Immune Cell Trafficking |
| **ACTB, AP1S2, BRD7, CEP170,** Cg, **COTL1**, Creb, **DNAJB9**, estrogen receptor, **FASN**, FSH, Gsk3, Hdac, Histone h3, Histone h4, **JMJD1C, KDM5C,** Lh, **MAF1, MXD1, MYSM1**, Nr1h, **PIAS1**, Rb, **RBBP8, RNASEH2A**, Smad2/3, **SMARCA4, SMC4**, Sos, **SYNE2, TAGLN2, TM4SF1, TSPAN13, XIST** | 29 | 22 | Cellular Development, Cellular Growth and Proliferation, Embryonic Development |

Bold type indicates that the genes are present in the RNA-seq data. Non-bolded type indicates that the genes are directly associated with the bold genes, which were obtained from the Ingenuity Knowledge Base.

**Supplementary Table 4.** List of biological processes from common DEGs amongst the three comparisons.

| **Biological process (Gene Ontology)** | **No. of gene** | **%*** | **Gene** |
|---|---|---|---|
| Biological adhesion (GO:0022610) | 3 | 1.9 | *RAP1A, NRAS, ROCK2* |
| Biological regulation (GO:0065007) | 8 | 5.1 | *BCL2L1, GNAI3, LIN7C, FAM60A, ATP1A1, YES1, CSNK1G3, DZIP3* |
| Cellular component organization or biogenesis (GO:0071840) | 27 | 17.1 | *UQCRB, RPL23A, ACTB, RAN, ATAD1, TOP1, CLIP1, RPS6, RPL23A, MYH9, RPL23A, ROCK2, TFIP11, ACTN4, DNM2, TMEM33, FAM96A, SMC4, NOP58, ACTN1, CSNK1G3, USO1, RICTOR, FLNB, DYNC1H1, DYNLT3, RPL7* |
| Cellular process (GO:0009987) | 74 | 46.8 | *RRAS2, SEPT7, AMD1, ARF4, PSMD12, SMAD5, BCL2L1, RAP1A, GNAI3, HTATSF1, CDC27, RNASEH2A, UQCRB, CHORDC1, RAB6A, LIN7C, GNB4, RPL23A, FAM60A, SYNE2, ACTB, RPS3, RAN, CEP135, NRAS, ATAD1, TOP1, ATP1A1, CLIP1, RASA2, RPS6, KIF20B, BSG, S100A16, DDX5, ZCCHC11, PDP1, RPL23A, PAPOLA, KLHL2, VPS35, MYH9, RPL23A, ROCK2, MOSPD3, MYO1C, TFIP11, STEAP3, YES1, HMGA1, ACTN4, MAGI3, DNM2, TMEM33, FAM96A, FKBP1A, PPIG, SMC4, POP7, RBBP8, NOP58, SUMO1, ZMYM1, ACTN1, HIAT1, CSNK1G3, USO1, RICTOR, DZIP3, FLNB, DYNC1H1, DYNLT3, PIP5K1C, RPL7* |
| Developmental process (GO:0032502) | 10 | 6.3 | *BCL2L1, SYNE2, UBE2D3, CLIP1, PTGR2, MYH9, ROCK2, MYO1C, YES1, MYL9* |
| Immune system process (GO:0002376) | 2 | 1.3 | *YES1, S100A16* |
| Localization (GO:0051179) | 28 | 17.7 | *RRAS2, ARF4, RAP1A, AP3S1, RAB6A, LIN7C, FAM60A, ACTB, RAN, NRAS, CLIP1, VPS35, MYH9, TMED5, MOSPD3, MYO1C, STEAP3, YES1, TMED2, DNM2, TMEM33, ERO1L, CSNK1G3, REEP3, USO1, DYNC1H1, DYNLT3, SDCBP* |
| Locomotion (GO:0040011) | 2 | 1.3 | *FAM60A, YES1* |
| Metabolic process (GO:0008152) | 66 | 41.8 | *AMD1, PSMD12, SMAD5, THAP5, GNAI3, ATP5B, HTATSF1, PQLC3, RNASEH2A, UQCRB, CD2AP, RPL23A, UBE2D3, HSP90B1, RPS3, RAN, ATAD1, TOP1, ATP1A1, CLIP1, RASA2, PDPR, RPS6, KIF20B, S100A16, DDX5, SSB, PTGR2, PDP1, RPL23A, GDPD5, PAPOLA, HRSP12, KLHL2, THOC2, RPL23A, TFIP11,* |

| | | | |
|---|---|---|---|
| | | | *EPC2, PPP1R12A, UPF3B, YES1, HMGA1, PRRG4, FAM96A, FKBP1A, PPIG, SMC4, POP7, LARP4, RBBP8, NOP58, SUMO1, ERO1L, ZMYM1, SND1, JMJD1C, NPM1, MXD1, CSNK1G3, REEP3, RICTOR, DZIP3, RBBP6, DYNLT3, PIP5K1C, RPL7* |
| Multicellular organismal process (GO:0032501) | 9 | 5.7 | *RAP1A, LIN7C, GNB4, NRAS, MYH9, MYO1C, MAGI3, MYL9, TAGLN2* |
| Reproduction (GO:0000003) | 1 | 0.6 | *DYNC1H1* |
| Response to stimulus (GO:0050896) | 11 | 7.0 | *BCL2L1, GNAI3, RNASEH2A, HSP90B1, RPS3, RASA2, YES1, HMGA1, CSNK1G3, RICTOR, DZIP3* |

*Percent of gene hit against total number of genes (158 DEGs).

**Supplementary Table 5.** List of biological processes from unique DEGs for the comparison between miNT blastocysts derived from depleted cells cultured in the absence of TSA and SCNT blastocysts derived from depleted cells cultured in the presence of TSA.

| Biological process (Gene Ontology) | No. of gene | %* | Gene |
|---|---|---|---|
| biological adhesion (GO:0022610) | 11 | 2.1 | *TNS1, CNTNAP1, ITGA5, CBLL1, COL17A1, COLGALT1, SCARB1, PLOD3, PLXNB2, NLGN2, RAP2C* |
| biological regulation (GO:0065007) | 42 | 8.0 | *PAK1, NDRG1, MAP3K7, TIMP1, TIMP2, AZIN1, AKT3, GNA11, CD63, NUCB2, ATAD2B, PLSCR1, SPNS1, RAP1GDS1, KRCC1, SLK, BAHD1, CBLL1, LATS1, SNAP23, PPRC1, CDC37,ADCY9 ,ESPL1 ,BCKDK ATXN2L, ACAD11, SLC30A1, EIF4A2, RAB8B, BOK, TANK, MAP2K2, PLXNB2, STX4, NLGN2, BAIAP2, PRKCZ, FKBP9, RPS6KA1, MASTL, GNAS* |
| cellular component organization or biogenesis (GO:0071840) | 66 | 12.6 | *CAPZA2, PAK1, SMC3, BRD3, HAT1, CLTB, DKC1, CFL2, DTNB, FLNC, POMT2, H3F3A, SNX10, BRWD1, KRT80, SF3B1, METTL17, PES1, LMNB2, H3F3A, STX12, XAB2, PLSCR1, CDC42EP1, TBC1D17, IPO4, BAHD1, EDC3, MTFR2, TUBGCP5, PRPF8, SASS6, HSPA4, COL17A1, SNAP23, PRPF6, NUP54, DDX6, ESPL1, SUPT6H, ATXN2L, GSN, SNX4, RAB8B, GNL1, WDR34, WASF1, TMOD3, CCT3, PLXNB2, STX4, ASF1A, NCAPD2, CBX3, NLGN2, BAIAP2, DNAH2, CCDC28B, KRT8, RPSA, TAF1B, ACTR3, KIF18B, CDK5RAP2, WASF2, DBN1* |
| cellular process (GO:0009987) | 239 | 45.6 | *CAPZA2, PAK1, ARHGDIA, SMC3, NDRG1, NEURL4, MAP3K7, TBK1, AFF1, ZFYVE16, BRD3, GSK3A, TIMP1, PPWD1, HAT1, CLTB, CALM, DKC1, NDUFB9, TIMP2, CFL2, SHROOM3, CTSA, DTNB, TNS1, FLNC, CNTNAP1, RASA1, POMT2, RPL29, OGT, H3F3A, SNX29, SNX10, NDNL2, RECQL4, RHOB, PCDH1, PIK3CA, AZIN1, AKT3, PDXK, SYMPK, BRWD1, GNA11, PIGN, CD63, EIF3L, SIRT7, CDKN3, BAZ2B, KRT80, PPP2CB, SF3B1, CDC42BPA, ABR, KAT5, EHD1, PES1, FOXO4, LMNB2, CTSD, H3F3A, SERINC2, RHOV, STX12, XAB2, ATAD2B, SENP6, VPS54, LOC531152, MOV10, PLSCR1, MTA2, UBE2J1, SPNS1, RAP1GDS1,* |

| | | | |
|---|---|---|---|
| | | | *CDC42EP1, LIG1, PFAS, SMAD7, RAVER1, KRCC1, SF3B2, SLK, TBC1D17, MLXIP, TRRAP, IPO4, CDC25A, ADA, BAHD1, EDC3, DDB1, CCNG1, RAB3IP, LOC515358, FTSJ1, CBLL1, SNRPB2, ATN1, MTFR2, TUBGCP5, PRPF8, MLXIPL, EIF3D, TUFM, CLTC, RPL8, SLC5A11, CTAGE5, PLEKHG3, CCNE2, DPF2, PPIL4, NPC1, RASSF5, LATS1, SASS6, LIN54, PLK1, CALM, COL17A1, RNPC3, SPTAN1, MTHFR, NR2C2AP, DAB2IP, PGM1, DNALI1, SNAP23, NUDC, PRPF6, NUP54, PPIA, B3GNT5, PPRC1, LGALS9, CSTF3, THOC1, CDC37, PIGK, RPL18, B3GNT3, ADCY9, MCM3, CLDN10, DDX6, APP, ESPL1, RHOV, SUPT6H, BCKDK, ATXN2L, ACAD11, RAB1B, EEF2, SLC30A1, TSEN54, ERCC2, PKIA, DSG2, GSN, EIF4A2, COLGALT1, SNX4, GLS, RAB8B, CNOT7, SLC25A6, EEF1G, GNL1, ABCA3, SUPT5H, EIF3B, TCEB1, BOK, TANK, BAZ2A, WDR34, FOXP4, SPIC, MYBL2, SOD1, EPN2, SCARB1, IGF2BP1, NCOA1, IQGAP3, YWHAQ, RCOR2, TMOD3, MAP2K2, PLXNB2, STX4, DSC2, TTK, SEC23A, ASF1A, TMEM189, NCAPD2, CBX3, BAIAP2, PRKCZ, DNAH2, ITPR1, KIF21A, CCDC28B, CLINT1, CCNF, KRT8, FKBP9, POLR2H, CDS2, RPSA, ATP5D, SLC25A1, SLC7A8, TAF1B, ACTR3, GEN1, CLIC4, PRKAR1A, KIF18B, CDK5RAP2, RPS6KA1, DTD1, ADRM1, RAPGEF1, RPS9, MIER1, DDX41, PARP1, MASTL, RAP2C, SLC7A4, DBN1, GNAS, FGGY* |
| developmental process (GO:0032502) | 34 | 6.5 | *PAK1, PCNXL3, NEURL4, ADAM9, ILF3, GSK3A, TNS1, FARP1, CNTNAP1, PCDH1, CDC42BPA, FOXO4, ZC3H10, MEF2D, VAT1,KRCC1 ,SLK ,DPF2 ,RASSF5, LATS1, IFRD1, B3GNT5, THOC1, B3GNT3, BOK, FOXP4, SPIC, IGF2BP1, TMOD3, MAP2K2, PLXNB2, NLGN2, CDK5RAP2, MIER1* |
| growth (GO:0040007) | 1 | 0.2 | *LATS1* |
| immune system process (GO:0002376) | 13 | 2.5 | *PRKAB1, TBK1, ILF3, ABCC1, TNS1, CNTNAP1, PTGS2, PLEKHG3, HSPA4, COL17A1, GPX2, GPX1, ITPR1* |
| localization (GO:0051179) | 69 | 13.2 | *MAL2, PAK1, ZFYVE16, CLCN3, ILF3, XPO1, AP3D1, CLTB, NDUFB9 ,SLC35A1, SHROOM3, ABCC1, TMED1, TTYH2, CNTNAP1, TRAM2, SNX29, SNX10, RHOB, EHD1, CHMP2B, SERINC2, RHOV, STX12, VPS54, LOC531152, PLSCR1, TBC1D17, IPO4, REEP5, AP1B1, CLTC, SLC5A11, CPNE2, NPC1, POLDIP3, NUP54 ,THOC1, PIGK, APP, RHOV, RAB1B, AP1B1, SLC29A1, SLC35A3, SNX4, DDHD1, RAB8B, ABCA3, WDR34, EPN2, IGF2BP1, MCFD2, SCAMP4, STX4, SEC23A, NLGN2, DNAH2, ITPR1, RPSA, PICALM, SLC7A8, ACTR3, CLIC4,* |

| | | | |
|---|---|---|---|
| | | | *CDK5RAP2, SEC14L1, RAP2C, SLC7A4, SLC46A1* |
| locomotion (GO:0040011) | 1 | 0.2 | *PAK1* |
| metabolic process (GO:0008152) | 217 | 41.4 | *CREG1, PAK1, SMC3, PRKAB1, MAP3K7, AFF1, PRODH, DHPS, ANXA5, BRD3, GSK3A, XPO1, TIMP1, PPWD1, HAT1, DKC1, NDUFB9, TIMP2, ATMIN, PRRC2A, SLC35A1, PFKFB4, CTSA, SRSF2, TNS1, CNTNAP1, RASA1, TRAM2, POMT2, RPL29, MVK, OGT, H3F3A, PHF3, RECQL4, PRPSAP1, ATP6V1A, MDFIC, PIK3CA, AZIN1, AKT3, PDXK, BRWD1, RBM10, GNA11, PGD, PIGN, EIF3L, SIRT7, CDKN3, BAZ2B, PPP2CB, SF3B1, ABR, ZNF335, KAT5, PIAS4, HSPB6, PES1, FOXO4, CTSD, GALT, H3F3A, ACO2, XAB2, ATAD2B, ZNF592, ANXA3, MOV10, MTA2, UBE2J1, RAP1GDS1, CDC42EP1, LIG1, PFAS, BTF3, SMAD7, VAT1, CBR4, RAVER1, SF3B2, SLK, MLXIP, TRRAP, SBNO2, CDC25A, RPLP1, ADA, BAHD1, EDC3, DDB1, ALAS1, ARL14EP, REEP5, GALNT1, FTSJ1, CBLL1, SNRPB2, GNE, ENPP5, MTFR2, PRPF8, MLXIPL, EIF3D, TUFM, RPL8, DPF2, TGM2, CAPN5, PPIL4, NPC1, LATS1SASS6, RPAP3, PTOV1, RNPC3, POLDIP3, PFKL, NR2C2AP, DAB2IP, PGM1, PRPF6, PCMTD1, PPIA, B3GNT5, PPRC1, RAD23B, CSTF3, THOC1, CDC37, PIGK, GPX2, RPL18, B3GNT3, ADCY9, MCM3, DDX6, TKT, SUPT6H, BCKDK, NPL, ACAD11, EEF2, GPX1, TSEN54, ERCC2, PKIA, TLE3, APH1A, SLC29A1, EIF4A2, POLRMT, SLC35A3, COLGALT1, KLK6, GLS, CNOT7, SLC25A6, EEF1G, GNL1, GALNT3, ABCA3, SUPT5H, EIF3B, TCEB1, TANK, CXXC1, BAZ2A, WDR34, FOXP4, SPIC, MYBL2, TAB2, GF2BP1, NCOA1, IDH3B, RCOR2, ESRP2, MAGT1, CCT3, MAP2K2, AGPS, PLXNB2, LHPP, NDUFA5, SMYD5, TTK, ASF1A, TMEM189, NCAPD2, CBX3, LSM14A, PRKCZ, KIF21A, FKBP9, POLR2H, CDS2, GAPDH, RPSA, ATP5D, SLC25A1, LARGE, TAF1B, GEN1, CLIC4, KIF18B, RPS6KA1, DTD1, ZNF408, SEC14L1, RPS9, MIER1, DDX41, PARP1, MASTL, GNAS, FGGY* |
| multicellular organismal process (GO:0032501) | 22 | 4.2 | *ADAM9, ILF3, CNTNAP1, PCDH1, DNAJC3, EHD1, LOC531152, RAB3IP, CLTC, PLEKHG3, LATS1, SNAP23, RAB8B, EPN2, IGF2BP1, TMOD3, PLXNB2, STX4, ITPR1, CLINT1, RAP2C, GNAS* |
| reproduction (GO:0000003) | 13 | 2.5 | *NEURL4, ADAM9, ILF3, NDNL2, CCNG1, TUBGCP5, CCNE2, LIN54, B3GNT5, B3GNT3, ESPL1, NCAPD2, CCNF* |
| response to stimulus (GO:0050896) | 46 | 8.8 | *PAK1, NDRG1, PRKAB1, MAP3K7, TIMP1, TIMP2, ABCC1, CNTNAP1, RASA1, RECQL4, AKT3, GNA11, CD63, PPP2CB, HSPB6, SPNS1, RAP1GDS1,* |

| | | | |
|---|---|---|---|
| | | | *CDC42EP1, LIG1, SMAD7, KRCC1, SLK, TRRAP, DDB1, MLXIPL, PLEKHG3, LATS1, RPAP3, DAB2IP, GPX2, ADCY9, SLC30A1, GPX1, BOK, TANK, SOD1, NCOA1, MCFD2, MAP2K2, BAIAP2, PRKCZ, ITPR1, RPS6KA1, PARP1, MASTL, GNAS* |

*Percent of gene hit against total number of genes (524 DEGs).

**Supplementary Table 6.** Effects of mitochondrial supplementation on the biological function of miNT blastocysts when compared with SCNT blastocysts derived from depleted cells cultured in the presence of TSA.

| Categories | Functions annotation | p-Value | Predicted State | Activation z-score | No. of genes |
|---|---|---|---|---|---|
| Carbohydrate metabolism | glycolysis of cells | 0.000747 | Increased | 2.412 | 10 |
| Cell cycle, DNA Replication, recombination, and repair | DNA recombination | 0.0000199 | Increased | 2.151 | 16 |
| | homologous recombination | 0.00000851 | Increased | 2.177 | 14 |
| | homologous recombination of cells | 0.0000011 | Increased | 2.514 | 13 |
| Cellular assembly and organization, cellular function and maintenance | microtubule dynamics | 0.00317 | Increased | 2.345 | 58 |
| | organization of cytoskeleton | 0.000165 | Increased | 2.639 | 73 |
| | organization of cytoplasm | 0.000775 | Increased | 2.639 | 76 |
| Cellular assembly and organization | formation of cytoplasmic aggregates | 0.000268 | Decreased | -2.176 | 6 |
| Embryonic development, organismal survival | death of embryo | 0.0000934 | Decreased | -2.028 | 14 |
| Gene expression | expression of mRNA | 0.00241 | Decreased | -2.414 | 14 |
| Gene expression, protein synthesis | translation of mRNA | 0.00154 | Decreased | -2.2 | 13 |
| Organismal survival | organismal death | 3.79E-14 | Decreased | -3.644 | 156 |
| | morbidity or mortality | 5.54E-14 | Decreased | -3.566 | 157 |
| Protein synthesis | modification of peptide | 0.0000429 | Decreased | -2.236 | 12 |
| | metabolism of peptide | 0.00137 | Decreased | -2.236 | 14 |

**Supplementary Table 7.** List of biological processes from unique DEGs from the comparison between miNT blastocysts derived from depleted cells cultured in the presence of TSA and SCNT blastocysts derived from depleted cells cultured in the presence of TSA.

| **Biological process (Gene Ontology)** | **No. of gene** | **%*** | **Gene** |
|---|---|---|---|
| biological regulation (GO:0065007) | 4 | 8.2 | *CD9, SLC34A2, SLC39A10, LSCR4* |
| cellular component organization or biogenesis (GO:0071840) | 5 | 10.2 | *NES, VBP1, RTN4, SIRT1, PLSCR4* |
| cellular process (GO:0009987) | 16 | 32.7 | *CD9, OGG1, PPP3CC, SLC25A11, NES, RABGGTA, LOC534630, UCHL3, VBP1, GCGR, ANKRD1, SLC34A2, RNF145, SLC39A10, SIRT1, PLSCR4* |
| developmental process (GO:0032502) | 6 | 12.2 | *PPP3CC, PBX3, DLX4, GCGR, ANKRD1, RTN4* |
| immune system process (GO:0002376) | 3 | 6.1 | *PPP3CC, GCGR, GLIPR2* |
| localization (GO:0051179) | 6 | 12.2 | *LOC534630, GCGR, SLC34A2, BCAP29, PLSCR4, NUP214* |
| metabolic process (GO:0008152) | 17 | 34.7 | *OGG1, PPP3CC, SLC25A11, RABGGTA, PBX3, DLX4, ACER3, LOC534630, SH3D21, UCHL3, PTPRA, CHSY1, DCPS, ANKRD1, PPA1, RNF145* |
| multicellular organismal process (GO:0032501) | 4 | 8.2 | *GCGR, ANKRD1, RTN4, CNN3* |
| reproduction (GO:0000003) | 2 | 4.1 | *GCGR, GLIPR2* |
| response to stimulus (GO:0050896) | 5 | 10.2 | *CD9, OGG1, PPP3CC, ACER3, GCGR* |

*Percent of gene hit against total number of genes (49 DEGs).

**Supplementary Table 8.** List of the significant differentially expressed genes from the comparison between miNT blastocysts derived from depleted and nondepleted cells. Fold changes are relative to miNT blastocysts derived from nondepleted cells.

| ID | Fold change | Regulation | FDR | Entrez Gene Name | Entrez gene ID | Location | Type(s) |
|---|---|---|---|---|---|---|---|
| *TMEM219* | 63.88560243 | Up | 0.0265 | transmembrane protein 219 | 506622 | Plasma Membrane | other |
| *APLP1* | 60.1000964 | Up | 0.0265 | amyloid beta precursor like protein 1 | 513154 | Extracellular Space | other |

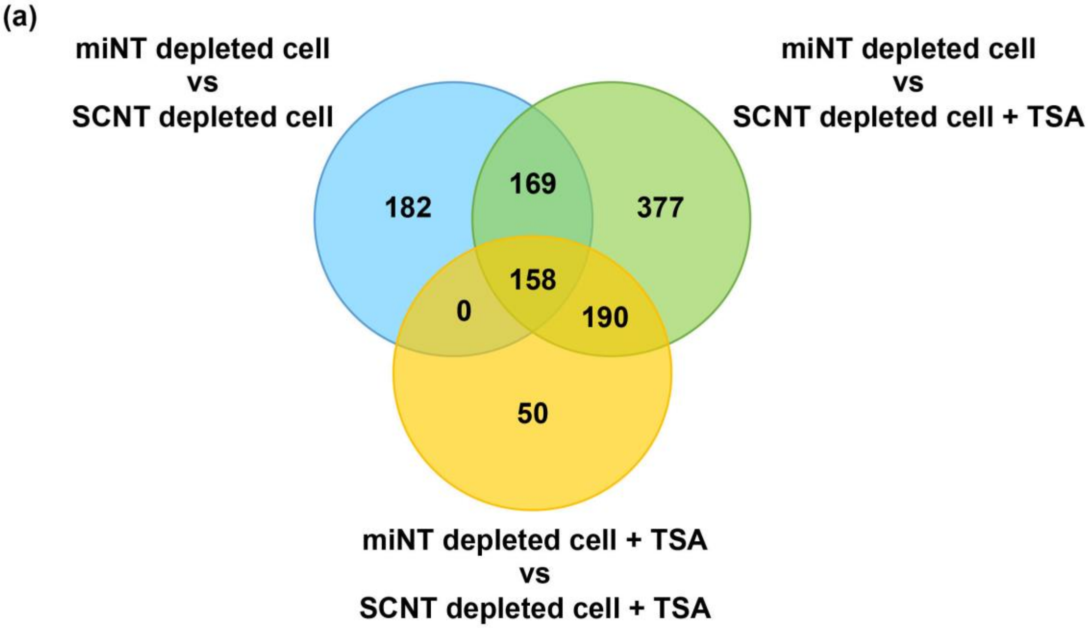


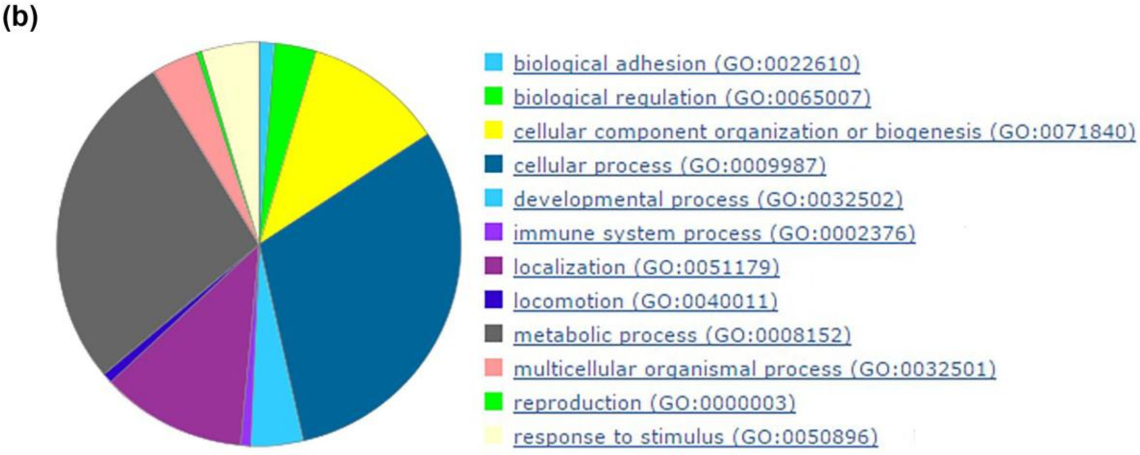


**Supplementary Figure 1.** Common DEGs amongst the three comparisons. (a) Venn diagram showing common DEGs amongst the comparisons between miNT and SCNT blastocysts derived from depleted cells cultured in the absence of TSA (blue); miNT blastocysts derived from depleted cells cultured in the absence of TSA and SCNT blastocysts derived from depleted cells cultured in the presence of TSA (green); and miNT and SCNT blastocysts derived from depleted cells cultured in the presence of TSA (Yellow) are shown. (b) PANTHER biological process classification.

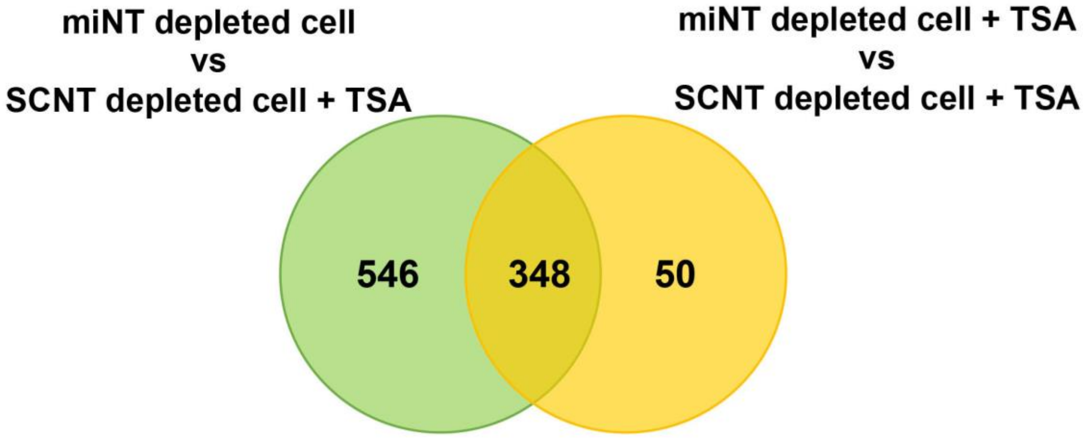


**Supplementary Figure 2.** Common DEGs between two comparisons. Venn diagram shows common and unique DEGs between comparisons of miNT blastocysts derived from depleted cells in the absence of TSA and SCNT blastocysts derived from depleted cells cultured in the presence of TSA (green); and miNT and SCNT blastocysts derived from depleted cells cultured in the presence of TSA (Yellow).
